# Reporting and methodological quality of systematic reviews underpinning clinical practice guidelines for low back pain: a meta-epidemiological study

**DOI:** 10.3389/fpain.2025.1704833

**Published:** 2025-12-03

**Authors:** Adam Khan, Will Roberts, Landon Frank, Anna Lillie, Trevor Torgerson, Aaron Pierce, Aaron Relic, Abdurrahman Khattab, Trevor Bright, Micah Hartwell, Matt Vassar

**Affiliations:** 1 Office of Medical Student Research, Oklahoma State University Center for Health Sciences, Tulsa, OK, United States; 2 Department of Pain Management and Anesthesiology, Baylor Scott & White Medical Center, Temple, TX, United States; 3 Department of Internal Medicine, Northeastern Health System, Tahlequah, OK, United States; 4 Department of Obstetrics & Gynecology, Oklahoma State University Medical Center, Tulsa, OK, United States; 5 Department of Head and Neck Surgery & Communication Sciences, Duke University School of Medicine, Durham, NC, United States; 6 Department of Anesthesiology, Oklahoma State University Medical Center, Tulsa, OK, United States; 7 Department of Internal Medicine, The University of Oklahoma School of Community Medicine, Tulsa, OK, United States

**Keywords:** low back pain, clinical practice guidelines, systematic reviews, methodological quality, evidence-based medicine

## Abstract

**Background:**

Low back pain (LBP) is the leading musculoskeletal disorder worldwide and a major cause of disability, health care utilization, and economic burden. Clinical practice guidelines (CPGs) aim to optimize care but depend heavily on systematic reviews (SRs). The reporting and methodological quality of SRs underpinning LBP CPGs remain unclear.

**Objectives:**

To conduct a meta-epidemiological assessment of the reporting and methodological quality of SRs cited in LBP CPGs and compare Cochrane vs. non-Cochrane reviews.

**Methods and design:**

Cross-sectional meta-epidemiological study. We identified English-language LBP CPGs published between 2017 and 2021 and extracted SRs underpinning therapeutic recommendations. Reporting quality was assessed using PRISMA and methodological quality using AMSTAR-2. Two reviewers performed masked, duplicate extraction with consensus resolution. Between-group comparisons used Wilcoxon rank-sum tests; prespecified subgroup analyses (by intervention domain) and an exploratory multivariable linear regression examined factors associated with PRISMA scores.

**Results:**

Eight CPGs cited 90 unique SRs. Mean PRISMA adherence was 83% (SD: 12.2); 39% of SRs met ≥90% of items. Mean AMSTAR-2 adherence was 79.3% (SD: 14.4); 24% were rated overall “high,” while 14% were “low/critically low.” Common deficits included protocol registration, justification of excluded studies, and assessment of small study/publication bias. Cochrane SRs (*n* = 22) had higher PRISMA (91% vs. 81%) and AMSTAR-2 (88% vs. 76%) scores than non-Cochrane SRs (both *p* < 0.001). Interventional technique SRs tended to have slightly lower PRISMA scores than pharmacologic SRs after adjustment, whereas noninvasive non-pharmacologic SRs were similar. In exploratory regression, higher AMSTAR-2 ratings and predominance of randomized trials were associated with higher PRISMA scores.

**Conclusions:**

SRs informing LBP CPGs show variable reporting and methodological quality with consistent shortfalls in protocol registration, exclusion justifications, and publication-bias assessment. Cochrane SRs outperformed non-Cochrane SRs yet comprised only a minority of the evidence base. Facilitating uptake of protocol registration, complete PRISMA-aligned reporting, transparent exclusion lists, and routine small-study bias assessment, alongside greater use of methodologically stronger SRs, could strengthen the evidentiary foundation of LBP guidelines.

## Introduction

Low back pain (LBP) is the most common musculoskeletal disorder worldwide and a leading cause of years lived with disability and health-care utilization, with substantial direct and indirect costs that disproportionately affect socio-economically disadvantaged groups (1). To mitigate this burden, clinical practice guidelines (CPGs), systematically developed statements intended to assist practitioner and patient decision-making, are expected to be grounded in the best available evidence and updated regularly (2). Because systematic reviews (SRs) synthesize primary studies and often sit near the apex of evidence hierarchies, they frequently underpin CPG recommendations. However, variability in SR reporting (PRISMA) and methodological quality (AMSTAR-2) has been documented across fields, raising concerns about the consistency and transparency of the evidence base that guides practice (3–6). This concern echoes broader meta-research showing the mass production of redundant or conflicted reviews that can mislead decision-making (7).

Beyond LBP, meta-research has repeatedly shown shortfalls that threaten review reliability: incomplete protocol registration, limited justification of excluded studies, and infrequent assessment of small study/publication bias (5, 6). Comparative work suggests that Cochrane SRs tend to exceed non-Cochrane SRs in methodological rigor and reporting clarity, yet they represent only a portion of the reviews informing clinical decisions (8, 9). At the trial level, selective outcome reporting and other departures from prespecified methods are well documented (e.g., COMPare audits), and initially impressive effects in highly cited studies often attenuate or reverse with subsequent evidence—signals that, if not handled transparently in SRs, can propagate into guideline recommendations (10–14). For guideline developers, these deficits matter because they can lead to overestimation of benefit, underrecognition of bias, and, ultimately, to stronger LBP recommendations than the underlying evidence justifies, especially for newer interventional or biologic approaches.

Despite extensive guideline activity for LBP, a key gap remains: to our knowledge, no study has mapped contemporary LBP CPG recommendations to their underpinning SRs and evaluated those SRs' reporting and methodological quality at the item level across multiple guidelines. Notably, prior appraisals of SRs on exercise therapy for chronic LBP have found very low overall confidence for most reviews, underscoring the need to examine the SRs that directly inform LBP guidelines (15). We therefore focused on CPGs published in a recent period of guideline and SR standardization (2017–2021), when PRISMA, AMSTAR-2, prospective registration (e.g., PROSPERO), and contemporary risk-of-bias methods were widely available, so that appraisal would be both contemporary and feasible across all linked SRs. Addressing this gap, we conducted a cross-sectional meta-epidemiological study to (1) identify recent LBP CPGs and the SRs cited for therapeutic recommendations; (2) appraise SR reporting using PRISMA and methodological quality using AMSTAR-2; and (3) compare Cochrane vs. non-Cochrane reviews. By linking CPG recommendations directly to SR quality, our study aims to clarify where transparency and rigor are strong, where they remain weakest (e.g., protocol registration, exclusion justifications, publication-bias assessment), and how these features may influence the reliability, strength, and patient-level applicability of LBP guideline recommendations.

## Methods

This study did not require institutional review board approval because it did not involve human subjects. A pre-specified protocol, including search strategy pilots, inclusion criteria, and extraction forms, was registered *a priori*, and all materials are openly available on the Open Science Framework (OSF) at https://osf.io/qw4dz/ (16). There were no deviations from the preregistered protocol; eligibility criteria, search strategy, data items, and analyses were performed as registered. We reviewed this meta-epidemiological study against the Murad and Wang 2017 reporting checklist (17) and made minor clarifications to improve transparency (for example, data items extracted, calibration procedures, and explicit cross-references to Supplementary Materials). The checklist and a line-by-line record of resulting manuscript edits are provided in Supplementary Table 6.

### Identification of clinical practice guidelines and systematic reviews

**Figure 1:**
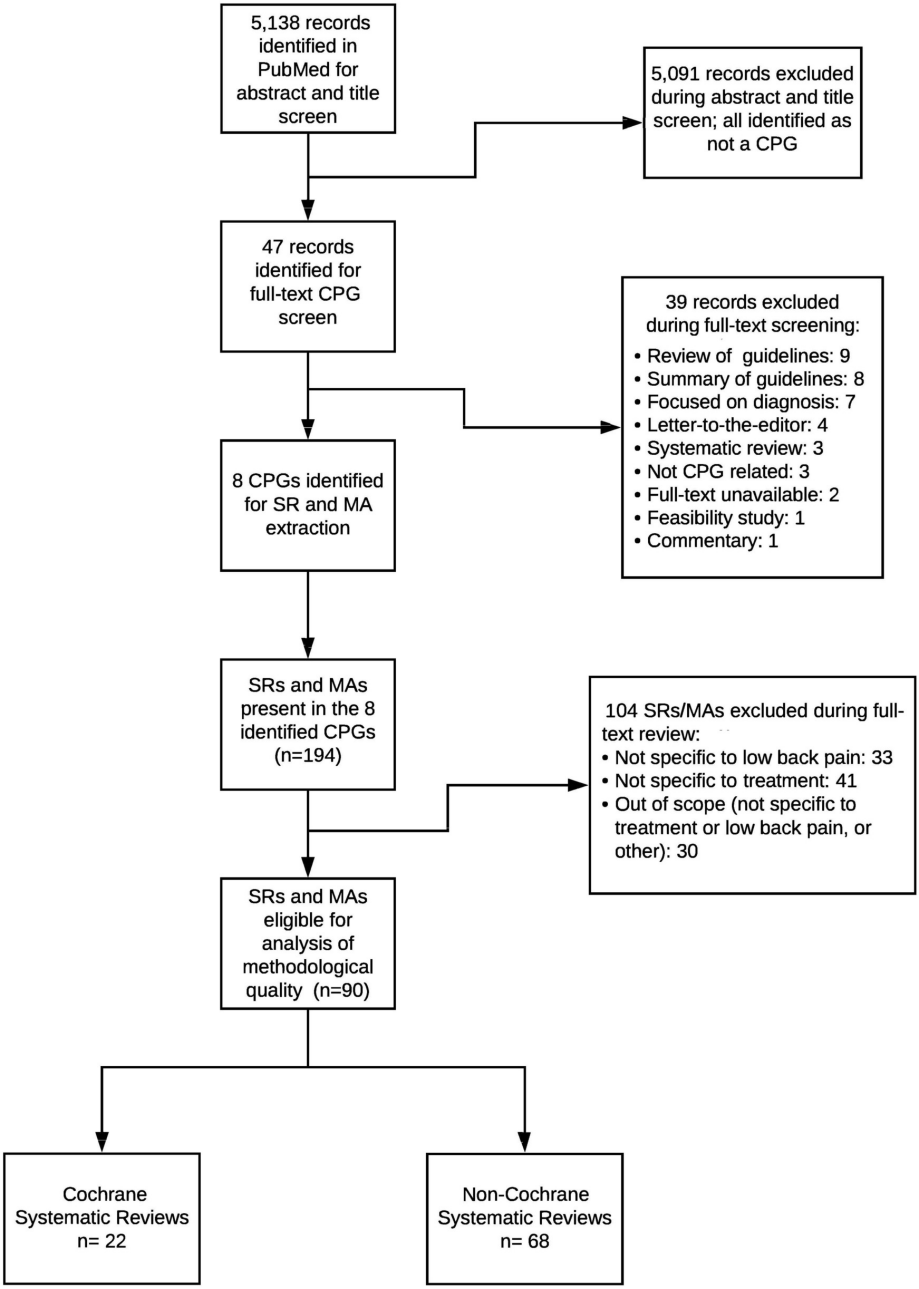
Flow diagram of the identification and selection of clinical practice guidelines (CPGs) and underpinning systematic reviews/meta-analyses (SRs/MAs). The PubMed search identified 5,138 records, of which 5,091 were excluded at title/abstract screening. Forty-seven full-text CPG records were assessed, and 39 were excluded for reasons such as non-CPG document type, diagnostic focus, or being out of scope. Eight CPGs were included and yielded 194 cited SRs/MAs, of which 104 were excluded at full-text review for not being specific to low back pain or treatment. Ninety SRs/MAs remained eligible for analysis, comprising 22 Cochrane and 68 non-Cochrane reviews.

We conducted a comprehensive PubMed search modeled on Canadian Agency for Drugs and Technologies in Health (CADTH) guideline filters to identify English-language clinical practice guidelines (CPGs) addressing therapeutic low back pain (LBP) interventions published between January 1, 2017, and June 3, 2021. Eligibility required meeting the Institute of Medicine definition of a CPG, primarily focusing on LBP management, and having full-text access. Titles, abstracts, and full texts were screened in duplicate in Rayyan, with disagreements adjudicated by a third reviewer. The 2017–2021 window (≈4.5 years) was prespecified to capture a recent period of LBP guideline development while preserving feasibility for item-level, dual-reviewer appraisal across multiple guidelines. We selected 2017 as the start point to align with a modern era of systematic review (SR) conduct and reporting (coinciding with the introduction of AMSTAR-2 in 2017 and broader uptake of PRISMA/PROSPERO and contemporary risk-of-bias methods), providing a pragmatic and conceptually coherent frame for appraisal. To mitigate missed items, we hand-screened reference lists of all eligible CPGs. The full PubMed search string and a line-by-line log of all records screened, with inclusion/exclusion reasons, are provided in Supplementary File 1 and Supplementary Table 5. Figure 1 presents a combined flow for CPG and SR screening; CPG-specific screening decisions and exclusion categories (for example, not an IOM-defined CPG/implementation or consensus document, outside the 2017–2021 window, non-English primary guideline, non-therapeutic LBP focus, duplicate or non-primary record, or lack of full text) are detailed in Supplementary Table 5.

From each included CPG, we screened the reference list for systematic reviews (SRs) using keyword and manual searches. SRs were eligible if they (i) were cited by the CPG as underpinning at least one therapeutic recommendation for LBP, (ii) met the PRISMA-P conceptual definition of a systematic review (clearly stated question, reproducible search, predefined eligibility criteria), (iii) were available in full text and in English, and (iv) addressed treatment or management of LBP. We included SRs with or without meta-analysis, including network meta-analyses. We excluded narrative or expert reviews, methodological overviews that did not fulfill SR criteria, reviews cited in the CPG but not linked to a specific therapeutic recommendation, and reviews primarily addressing non-LBP conditions.

### Data extraction and scoring

After duplicate removal, two masked reviewers independently extracted study characteristics and evaluated reporting quality with the 27-item PRISMA checklist and methodological quality with the 16-item AMSTAR-2 tool. Before formal extraction, reviewers completed a pilot calibration on a small set of SRs to harmonize scoring rules, including explicit decision criteria for when to assign “partial yes” vs. “no” for each PRISMA and AMSTAR-2 item. For each CPG, we recorded title, issuing organization, year, scope, and the linkage between recommendations and underpinning SRs. For each SR, we extracted citation (journal, year), Cochrane vs. non-Cochrane status, intervention domain (pharmacologic, nonpharmacologic, interventional), clinical population focus (e.g., with or without radiculopathy), study-design composition (e.g., proportion randomized trials), number of included primary studies and participants (when reported), funding source and author conflict-of-interest statements, protocol registration or published protocol (e.g., PROSPERO ID), the risk-of-bias tool used by the SR, whether small-study/publication-bias was assessed, and item-level PRISMA and AMSTAR-2 scores. Because the unit of analysis was the SR, risk-of-bias within primary studies was not collected; instead, methodological quality of SRs was appraised with AMSTAR-2. We did not calculate a formal inter-reviewer agreement statistic; instead, reviewers underwent pilot calibration and reconciled all discrepancies by consensus, which we acknowledge as a limitation. Because item-level judgments and skewed item prevalence can distort kappa, we prioritized masked duplicate assessment followed by consensus after calibration.

Each PRISMA/AMSTAR-2 item was scored as yes = 1, partial yes = 0.5, or no = 0 using a rubric refined during a pilot calibration exercise. Examples of partial yes included: PRISMA “protocol and registration” when a protocol was mentioned but not publicly accessible (no URL/identifier), vs. no when no protocol was mentioned; PRISMA “structured abstract” when most required elements were present but registration or funding was missing, vs. no when multiple required elements were absent; AMSTAR-2 “list of excluded studies and justification” when a list was provided without explicit reasons, vs. no when neither a list nor reasons were provided; and AMSTAR-2 “study selection and data extraction in duplicate” when duplication was reported for one process (selection or extraction) but not both, vs. no when neither process was duplicated. A third reviewer was available but not required. Cochrane authorship was verified through the Cochrane Library. Item-level and study-level data are summarized in the Supplement (Supplementary Tables S2–S4), and the screened-record log appears in Supplementary Table 5.

We appraised SR reporting using the PRISMA 2009 checklist because most underpinning SRs pre-dated the March 2021 PRISMA 2020 update. Using the contemporaneous checklist ensured fair evaluation against standards in place when the SRs were written; PRISMA 2009 and 2020 (18, 19) have substantial item overlap, and switching checklists mid-study would require re-calibration outside our registered protocol. Item-level PRISMA and AMSTAR-2 data are provided in Supplementary Tables 2 and Table 3, overall AMSTAR-2 ratings in Supplementary Table 4**,** and the record-level screened CPG log in Supplementary Table 5.

### Secondary analysis and statistics

We compared Cochrane vs. non-Cochrane SRs using the Wilcoxon rank-sum (Mann–Whitney) test and report exact *P*-values. Descriptive statistics summarized all scores, and non-parametric methods were used where appropriate. In addition, we conducted prespecified subgroup comparisons (by Cochrane status and intervention domain) and an exploratory multivariable linear regression with PRISMA score (continuous; percentage of 27 items met, scaled 0–100) as the outcome and AMSTAR-2 rating (categorical), Cochrane status, intervention domain (pharmacologic, nonpharmacologic, interventional), and study-design composition (predominantly randomized trials vs. mixed) as predictors. Robust standard errors were used, and model diagnostics (linearity and residual patterns) were examined. Full coefficient estimates with 95% CIs are provided in Supplementary Table 1. Two-sided *α* was set at 0.05. Analyses were performed with Stata 16.1.

## Results

The search strategy yielded 5,138 records. After screening, eight LBP CPGs (20–27) met inclusion (Figure 1). Among the records screened, the most frequent reasons for CPG exclusion were non-eligible document type (e.g., implementation framework or consensus statement rather than an IOM-defined CPG), publication outside the 2017–2021 window, and non-English primary guideline documents; additional exclusions reflected scope mismatch (non-therapeutic LBP) and duplicate or non-primary records. The complete record-level log of CPG screening decisions appears in Supplementary Table 5. From the eight included CPGs, we identified 194 SRs/meta-analyses; after excluding 104 that were out of scope (not specific to low back pain, not specific to treatment, or other), 90 unique SRs addressing therapeutic interventions formed the analytic sample. Publication years spanned 2002–2020; most reviews examined interventional pain techniques or non-pharmacological, non-invasive therapies (37% each). Sixty-eight SRs (76%) analyzed predominantly randomized trials; 87 (97%) declared conflicts of interest, yet 39% omitted funding sources. Detailed study-level characteristics are provided (Supplementary Table 1 for regression covariates; Supplementary Table 5 for the screened CPG log). The median number of primary studies included per SR was 15 (interquartile range: 9–27), with pooled sample sizes spanning 120–12,400 participants. Formal network meta-analysis was uncommon, appearing in only 3% of reviews, whereas 41% relied exclusively on qualitative synthesis. A temporal analysis showed that 58% of the included SRs were published in 2015–2020, reflecting accelerating research output in this field. The characteristics of the eight included CPGs are summarized (Table 1).

**Table 1:** Characteristics of the included clinical practice guidelines.

Clinical practice guideline	Author	Year of publication	PMID	Geographical region	References per guideline
Best Practices for Chiropractic Management of Patients with Chronic Musculoskeletal Pain: A Clinical Practice Guideline	Hawk et al.	2020	32749874	USA	103
Comprehensive Evidence-Based Guidelines for Facet Joint Interventions in the Management of Chronic Spinal Pain: American Society of Interventional Pain Physicians (ASIPP) Guidelines	Manchikanti et al.	2020	32503359	USA	687
Evidence-Based Recommendations on the Pharmacological Management of Osteoarthritis and Chronic Low Back Pain: An Asian Consensus	Yabuki et al.	2019	31382324	Asia (China, Japan, Taiwan, Korea), Australia, Canada	58
JAMA Clinical Guidelines Synopsis Treatment of Low Back Pain	Wenger et al.	2017	28829855	USA	10
Noninvasive Treatments for Acute, Subacute, and Chronic Low Back Pain: A Clinical Practice Guideline From the American College of Physicians	Qaseem et al.	2017	28192789	USA	182
Nonsurgical treatments for patients with radicular pain from lumbosacral disc herniation	Lee et al.	2019	31201860	South Korea	94
Responsible, Safe, and Effective Use of Biologics in the Management of Low Back Pain: American Society of Interventional Pain Physicians (ASIPP) Guidelines	Navani et al.	2019	30717500	USA	353
Spinal Manipulative Therapy and Other Conservative Treatments for Low Back Pain: A Guideline From the Canadian Chiropractic Guideline Initiative	Bussieres et al.	2018	29606335	Canada	169

### PRISMA

Overall mean PRISMA adherence was 83.0% (SD: 12.2) (Supplementary Figure 2). Thirty-five reviews (39%) fulfilled ≥90% of checklist items, whereas ten (11%) met <70%, indicating a wide distribution of reporting completeness. Highest-scoring items were rationale (Item 3) and additional analyses (item 16). Item-level performance is detailed in Supplementary Table 2. Persistent shortcomings were observed for protocol registration (item 5, 54%) and explicit objectives (item 4, 59%), echoing patterns documented across pain-medicine subspecialties. Item-level PRISMA performance is detailed in Supplementary Table 2, with distribution plots in Supplementary Figure 2.

### AMSTAR-2

Mean AMSTAR-2 adherence was 79.3% (SD: 14.4) (Supplementary Figure 3a). Twenty-two reviews (24%) achieved an overall “high” rating, whereas 14% were graded “low” or “critically low.” Critical weaknesses clustered around justification of excluded studies (item 7, 46%), reporting funding of included studies (item 10, 42%), and publication-bias assessment (item 15, 47%) (Supplementary Table 3). Four reviews (4%) were rated “critically low,” 10 “low,” 54 “moderate,” and 22 “high” (Supplementary Table 4). Methodological quality improved only modestly over the study period, underscoring the need for continued emphasis on transparent, protocol-driven review conduct. Item-level AMSTAR-2 performance is shown in Supplementary Table 3; overall AMSTAR-2 ratings for each systematic review appear in Supplementary Table 4. Distribution and subgroup plots are provided in Supplementary Figure 3.

### Cochrane vs. Non-Cochrane Reviews

Of the 90 studies included in our analysis, we found 22 to be conducted by a Cochrane group (24.4%) and 68 to be conducted by a non-Cochrane group (75.6%). Regarding the Cochrane conducted SRs, adherence to the PRISMA reporting guidelines was high with a mean PRISMA completion percentage of 90.6% (SD = 2.6%). The non-Cochrane SR's adherence was lower with a mean PRISMA completion percentage of 80.5% (SD = 13.1%; Supplementary Table 1). This association between Cochrane SRs and non-Cochrane SRs remained consistent when evaluating the studies' methodological quality with the AMSTAR-2 tool. Cochrane conducted SRs had a mean AMSTAR-2 completion percentage of 88.2% (SD = 6.4%) while non-Cochrane conducted SRs had a mean AMSTAR-2 completion percentage of 76.4% (SD = 15.1%; Supplementary Table 3). A Mann–Whitney *U* test confirmed that there was a significant difference between Cochrane conducted and non-Cochrane conducted PRISMA (*z* = −4.51; *p* = 6.6 × 10^−6^) and AMSTAR-2 completion (*z* = −4.27; *p* = 1.9 × 10^−5^).

### Domain differences

In prespecified subgroup comparisons and in an exploratory multivariable model, interventional pain technique SRs tended to have lower PRISMA scores than pharmacologic SRs after adjustment (*p* = 0.056), while noninvasive non-pharmacologic SRs were similar to pharmacologic SRs; domain-level differences in AMSTAR-2 were modest. Full unadjusted and adjusted regression coefficients with 95% confidence intervals are reported in Supplementary Table 1.

### Exploratory analysis

In multivariable models, higher AMSTAR-2 ratings and reviews that predominantly included randomized trials were associated with higher PRISMA scores. Interventional pain technique reviews tended to score lower than drug therapy reviews after adjustment (*p* = 0.056), while noninvasive non-pharmacologic reviews were similar to drug therapy. Reporting a conflict-of-interest statement was associated with higher PRISMA scores, whereas declaring no funding was associated with lower scores. Full unadjusted and adjusted estimates with 95% CIs are provided in Supplementary Table 1.

## Discussion

This cross-sectional meta-epidemiological study found variable adherence to PRISMA and AMSTAR-2 among the 90 SRs informing therapeutic LBP recommendations; consistent with evidence from oncology and orthodontics, Cochrane reviews delivered markedly higher reporting completeness and methodological rigor (8, 9) yet constituted only one-quarter of the evidence base, meaning guideline panels that do not prioritize these higher-quality sources risk basing recommendations on less robust evidence (21). Deficiencies clustered around protocol registration, justification of excluded studies, disclosure of funding sources, and assessment of publication bias, items repeatedly identified as weak points across medical specialties (4, 22), and these specific gaps matter for guideline reliability because, without a protocol, an exclusion list with reasons, or a check for small-study/publication bias, panels have limited ability to judge whether an apparently favorable pooled effect is vulnerable to selective reporting. Our multiple-regression analysis confirmed a positive association between methodological quality and reporting completeness, reinforcing that rigorous methods translate into clearer reporting; with the rapid expansion of interventional pain medicine and non-pharmacological modalities for LBP, facilitating uptake of established reporting and methods standards may help panels avoid issuing strong recommendations on the basis of unstable effects and may discourage premature uptake of costly or invasive options (23). Taken together with prior meta-research in other fields, these convergent patterns suggest that challenges in protocol registration, exclusion justification, and publication-bias assessment are systemic rather than LBP-specific. Limited assessment of publication bias has direct implications for guideline formulation: when small-study or selective-publication effects are not examined, pooled estimates may be inflated or less precise, particularly in domains dominated by small or early-phase trials, so “positive” findings can be over-represented and the strength of recommendations inadvertently overstated. In several SRs, formal tests were infeasible (for example, too few included studies or substantial heterogeneity), but explicit acknowledgement of this constraint and use of sensitivity analyses could improve confidence in the underlying evidence (28).

Implications for guideline developers and clinicians follow directly from these observations. When SRs omit protocol registration or exclusion justifications, or do not consider small-study/publication bias, certainty in pooled estimates may be overstated, increasing the likelihood that CPGs present a strong or unqualified recommendation where a conditional recommendation would be more appropriate—especially for newer interventional techniques, biologic approaches, or device-based therapies. Facilitating uptake of protocol registration, reproducible study selection with reasons, and routine consideration of small-study bias could support clearer strength-of-recommendation grading and better alignment of coverage and clinical decisions with the actual robustness of the evidence. Beyond LBP, prospective audits have documented selective outcome reporting and protocol deviations in trials (COMPare), and highly cited clinical studies often show attenuated or contradicted effects over time, indicating that early, incompletely reported evidence can overstate benefits (10–14). These ecosystem-level findings reinforce our item-level results and underscore why SRs that inform LBP guidelines benefit from transparent protocols, explicit exclusion justifications, and routine publication-bias assessment.

Reporting and methodological quality varied little across therapeutic domains. Interventional technique SRs showed slightly lower PRISMA adherence than pharmacologic SRs after adjustment, whereas noninvasive non-pharmacologic SRs were comparable. Consistent item-level weaknesses—protocol registration, justification of excluded studies, and assessment of publication bias—were observed across domains, suggesting that review conduct and transparency practices**,** rather than the therapeutic area itself, could account for quality differences.

Context from other specialties suggests our mean adherence levels are on the higher side (5, 28). For example, rehabilitation journals have reported mean PRISMA adherence around 60% (5), and in oncology, AMSTAR-2 confidence has frequently been rated “critically low” for the majority of SRs (6, 29). Comparable appraisals in cardiology likewise report predominantly low/critically low AMSTAR/AMSTAR-2 ratings and frequent under-reporting of protocol registration and publication-bias assessment (30). We therefore interpret our higher averages as reflecting (i) sample composition that included several Cochrane SRs and (ii) our prespecified allowance for “partial yes” when elements were present but incomplete, rather than indicating uniformly superior SR quality in LBP; importantly, protocol registration, justification of exclusions, and publication-bias assessment still show room for improvement. Overall, we judge our confidence in the direction and robustness of these findings to be moderate, based on (i) consistency across two independent tools (PRISMA, AMSTAR-2); (ii) the magnitude and coherence of Cochrane vs. non-Cochrane differences across items; and (iii) agreement between unadjusted comparisons and exploratory adjusted analyses. While our cross-sectional design and English-language focus limit certainty, transparency measures (open protocol, full search string, line-by-line screening log, and item-level extractions) support reproducibility and independent appraisal.

In practice, guideline panels can place relatively more weight on recommendations supported by higher-quality reviews (for example, Cochrane) and apply additional caution when guidance relies on non-Cochrane SRs with incomplete methods reporting. In such cases, certainty may be lower, and clinicians could consider more conservative implementation, especially for newer interventional techniques, until corroborating evidence emerges. Policymakers may wish to weight SRs with transparent protocols, exclusion justifications, and bias assessments more heavily; qualify recommendation strength when these elements are absent; and request clear conflict-of-interest and funding disclosures before broad implementation or coverage decisions.

### Strengths and limitations

Strengths include adherence to a prospectively registered protocol, duplicate masked extraction, and public sharing of all materials via OSF, which together enhance transparency and reproducibility. We also provide the full PubMed search string and a line-by-line log of all records screened with inclusion/exclusion reasons in Supplementary File 1 and Supplementary Table 5, supporting replication and future updates. Further, in response to the Murad–Wang reporting checklist (17), we refined several reporting elements (for example, explicit listing of extracted variables and justification of consensus procedures). The specific changes prompted by the checklist review are detailed in Supplementary Table 6.

Limitations mirror prior meta-research. This cross-sectional design cannot assess temporal trends. Our CPG search used PubMed with CADTH-style filters and was restricted to English-language primary guidelines, so some non-English or non-indexed CPGs may have been missed despite hand-screening reference lists. This English-language restriction may bias the sample toward anglophone, higher-income settings and underrepresent non-English guidance, limiting generalizability. PRISMA/AMSTAR-2 scoring retains some subjectivity, particularly for partial-yes determinations, and we did not calculate a formal inter-rater reliability statistic; instead, reviewers underwent pilot calibration and reconciled all discrepancies by consensus. Because prevalence- and bias-dependent effects can misrepresent agreement on skewed, item-level judgments, we did not compute Cohen's kappa; instead, we prioritized consensus after calibration and provide item-level decisions to permit independent calculation of agreement if desired. To enable independent appraisal, complete item-level extractions and screening logs are provided (Supplementary Tables 2–S5). The prespecified window (January 2017–June 2021) preserved feasibility for item-level, dual-reviewer appraisal across multiple guidelines; although additional CPGs/SRs have since appeared, the direction and magnitude of our main findings are unlikely to change materially with incremental additions. Finally, because most SRs were published before March 2021, we used PRISMA 2009 for a contemporaneous appraisal while acknowledging that future updates could adopt PRISMA 2020 (18, 19). To enhance coverage, future updates will also expand beyond PubMed to additional bibliographic databases (e.g., Embase) and guideline repositories.

## Conclusion

We recommend facilitating uptake of established methods in SRs that inform LBP guidelines—protocol registration, complete PRISMA-aligned reporting, transparent justification of exclusions, and routine assessment of small-study/publication bias. Guideline panels may wish to weigh evidence from methodologically stronger SRs (for example, Cochrane) more heavily and to qualify recommendation strength when key elements are absent. These steps could improve the evidentiary foundation of LBP CPGs and promote more reliable, patient-centered care.

## Data Availability

The datasets presented in this study can be found in online repositories. The names of the repository/repositories and accession number(s) can be found in the article/Supplementary Material.
