# Supplementary material for: Reporting and methodological quality of systematic reviews underpinning clinical practice guidelines for low back pain: a meta-epidemiological study
Source: Front Pain Res (Lausanne). 2025 Dec 3;6:1704833. doi: 10.3389/fpain.2025.1704833 (PMC12708511; doi:10.3389/fpain.2025.1704833)
Supplement: Supplementary file 1 [file Table1.docx]

:((((((low back pain[MeSH Terms]) OR (back pain[MeSH Terms])) OR (back pain with radiation[MeSH Terms])) OR (back pain without radiation[MeSH Terms])) OR (back pain, low[MeSH Terms])) AND (Clinical pathway[mh] OR Clinical protocol[mh] OR Consensus[mh] OR Consensus development conferences as topic[mh] OR Critical pathways[mh] OR Guidelines as topic [Mesh:NoExp] OR Practice guidelines as topic[mh] OR Health planning guidelines[mh] OR guideline[pt] OR practice guideline[pt] OR consensus development conference[pt] OR consensus development conference, NIH[pt] OR position statement*[tiab] OR policy statement*[tiab] OR practice parameter*[tiab] OR best practice*[tiab] OR standards[ti] OR guideline[ti] OR guidelines[ti] OR ((practice[tiab] OR treatment*[tiab]) AND guideline*[tiab]) OR CPG[tiab] OR CPGs[tiab] OR consensus*[tiab] OR ((critical[tiab] OR clinical[tiab] OR practice[tiab]) AND (path[tiab] OR paths[tiab] OR pathway[tiab] OR pathways[tiab] OR protocol*[tiab])) OR recommendat*[ti] OR (care[tiab] AND (standard[tiab] OR path[tiab] OR paths[tiab] OR pathway[tiab] OR pathways[tiab] OR map[tiab] OR maps[tiab] OR plan[tiab] OR plans[tiab])) OR (algorithm*[tiab] AND (screening[tiab] OR examination[tiab] OR test[tiab] OR tested[tiab] OR testing[tiab] OR assessment*[tiab] OR diagnosis[tiab] OR diagnoses[tiab] OR diagnosed[tiab] OR diagnosing[tiab])) OR (algorithm*[tiab] AND (pharmacotherap*[tiab] OR therap*[tiab] OR treatment*[tiab] OR intervention*[tiab]))) ) OR (back pain, lower[MeSH Terms]).
