# Supplementary material for: Reporting and methodological quality of systematic reviews underpinning clinical practice guidelines for low back pain: a meta-epidemiological study
Source: Front Pain Res (Lausanne). 2025 Dec 3;6:1704833. doi: 10.3389/fpain.2025.1704833 (PMC12708511; doi:10.3389/fpain.2025.1704833)
Supplement: Supplementary file 2 [file Table2.docx]

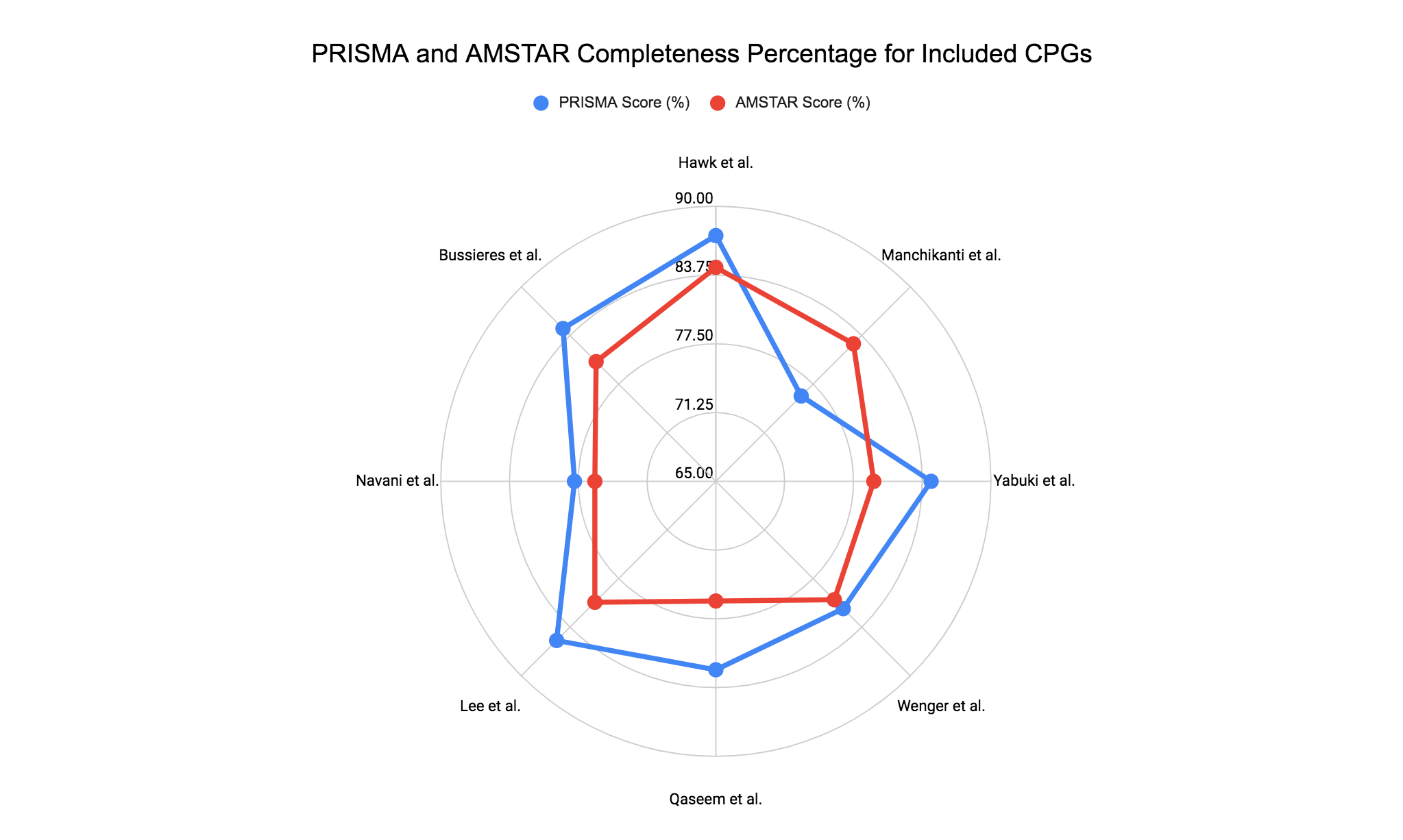


Supplementary Figure 2. Representation of overall AMSTAR and PRISMA completeness scores (percentages) for the included clinical practice guidelines (n=8) listed by the author of the guideline
