# Supplementary material for: Reporting and methodological quality of systematic reviews underpinning clinical practice guidelines for low back pain: a meta-epidemiological study
Source: Front Pain Res (Lausanne). 2025 Dec 3;6:1704833. doi: 10.3389/fpain.2025.1704833 (PMC12708511; doi:10.3389/fpain.2025.1704833)
Supplement: Supplementary file 3 [file Table3.docx]

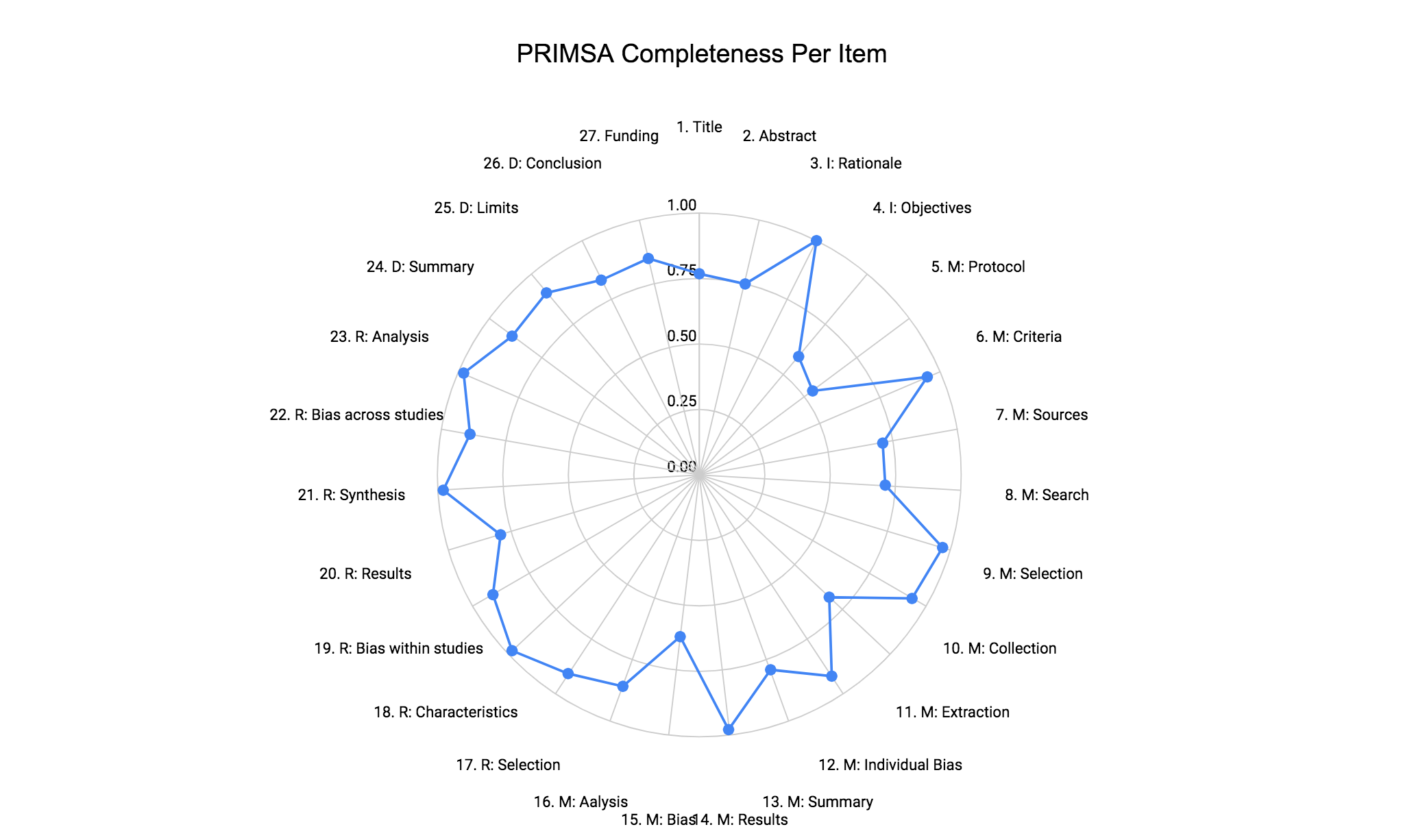


Supplementary Figure 3a: Mean score of the systematic reviews (n=90) in the included clinical practice guideline (n=8) for each PRISMA item. I, Introduction; M, Methods; R, Results; D, Discussion


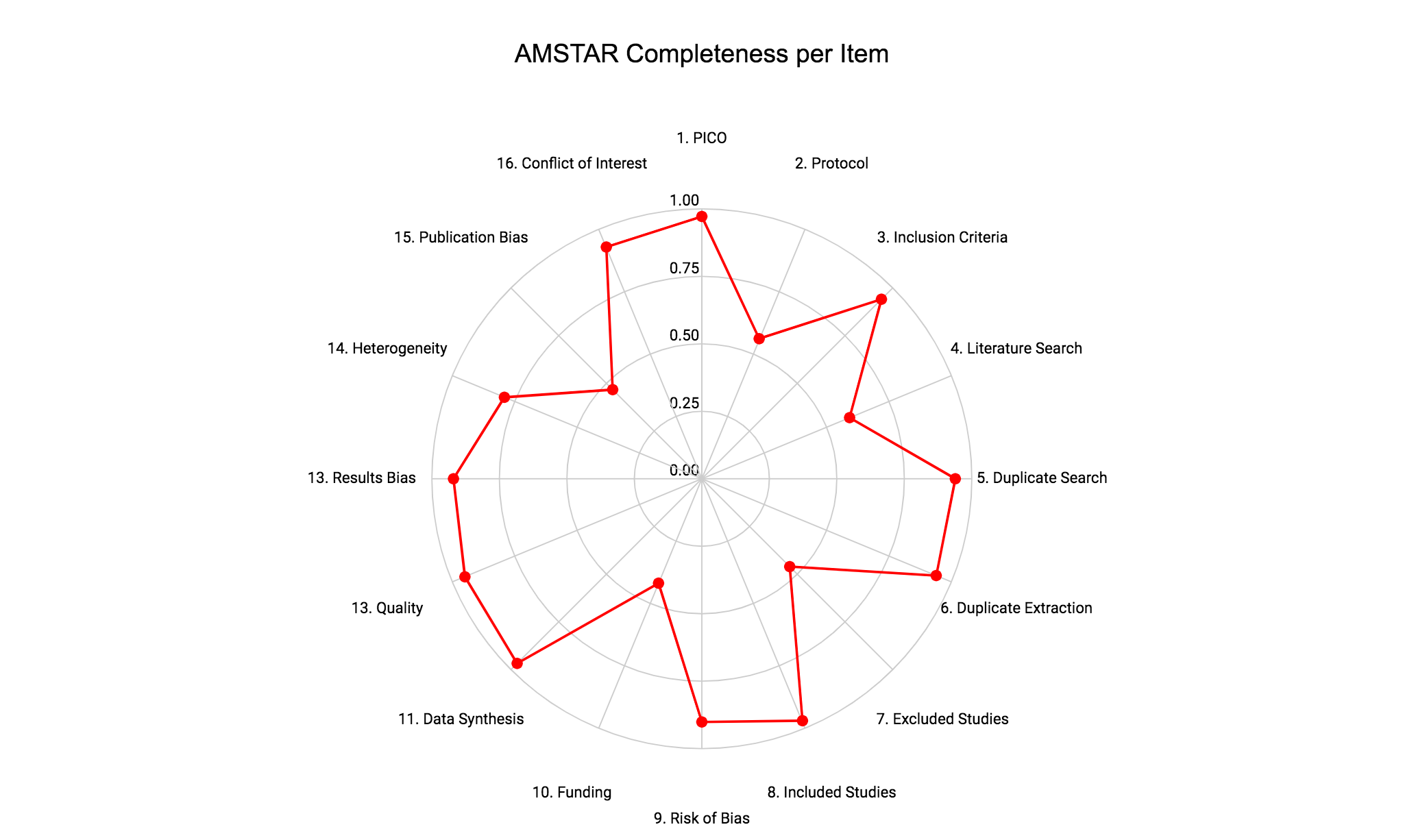


Supplementary Figure 3b: Mean score of the systematic reviews (n=90) in the included clinical practice guideline (n=8) for each AMSTAR item.
