# Supplementary material for: Reporting and methodological quality of systematic reviews underpinning clinical practice guidelines for low back pain: a meta-epidemiological study
Source: Front Pain Res (Lausanne). 2025 Dec 3;6:1704833. doi: 10.3389/fpain.2025.1704833 (PMC12708511; doi:10.3389/fpain.2025.1704833)
Supplement: Supplementary file 4 [file Table4.docx]

| **Supplementary Table 1. Multiple-Regression Analysis of Systematic Review Characteristics** | | | | | | | | |
| --- | --- | --- | --- | --- | --- | --- | --- | --- |
| **Covariables** | **No., (%) of Articles (n=90)** | **Unadjusted model**  **Coef. (SE)** | **t-value** | ***P*** | **Adjusted model**  **Coef., (SE)** | **Standardized**  **Coef** | **t-value** | ***P*** |
| **Year of Publication** | | | | | | | | |
| Before 2010 | 5 (5.56) | 1 [Ref] | - | - | 1 [Ref] | [Ref] | - | - |
| After 2010 | 85 (94.44) | 17.76, (5.34) | 3.33 | **<.001** | 5.3 (4.09) | 0.1 | 1.3 | 0.199 |
| **Intervention Type** | | | | | | | | |
| Drug Therapy | 20 (22.22) | 1 [Ref] | - | - | 1 [Ref] | [Ref] | - | - |
| Interventional Pain Technique | 33 (36.67) | -8.4 (3.25) | -2.59 | **0.011** | -4.29 (2.21) | -0.17 | -1.94 | 0.056 |
| Noninvasive Nonpharmacological Therapy | 33 (36.67) | 2.15 (3.25) | 0.66 | 0.509 | 1.24 (2.05) | 0.05 | 0.61 | 0.547 |
| Other (Device, Behavioral, Combo) | 4 (4.44) | 1.35 (6.27) | 0.22 | 0.83 | 2.09 (4.14) | 0.04 | 0.51 | 0.615 |
| **Conflict of Interest** | | | | | | | | |
| Statement Not Reported | 3 (3.33) | 1 [Ref] | - | - | 1 [Ref] | [Ref] | - | - |
| Statement Reported | 87 (96.67) | 24.82, (6.72) | 3.69 | **<.001** | 14.7 (5.28) | 0.22 | 2.78 | **0.007** |
| **Design of Included Studies** | | | | | | | | |
| Primarily Non-RCTs | 30 (33.33) | 1 [Ref] | - | - | 1 [Ref] | [Ref] | - | - |
| Primarily RCTs | 60 (66.67) | 8.86, (2.58) | 3.43 | **0.001** | 4.63 (1.81) | 0.18 | 2.57 | **0.012** |
| **AMSTAR-2 Rating** | | | | | | | | |
| Critically Low | 4 (4.44) | 1 [Ref] | - | - | 1 [Ref] | [Ref] | - | - |
| Low | 10 (11.11) | 21.69, (5.23) | 4.15 | **<.001** | 16.27 (5.02) | 0.42 | 3.24 | **0.002** |
| Moderate | 54 (60.00) | 32.37, (4.58) | 7.06 | **<.001** | 27.27 (4.11) | 1.1 | 6.63 | **<.001** |
| High | 22 (24.44) | 40.09, (4.81) | 8.34 | **<.001** | 32.28 (4.5) | 1.14 | 7.17 | **<.001** |
| **Funding** | | | | | | | | |
| No Funding Statement | 35 (38.89) | 1 [Ref] | - | - | 1 [Ref] | [Ref] | - | - |
| No Funding Received | 17 (18.89) | -8.44, (3.36) | -2.51 | **0.014** | -6.76 (2.43) | -0.22 | -2.79 | **0.007** |
| Industry | 2 (2.22) | -9.42, (8.26) | -1.14 | 0.257 | 1.26 (5.74) | 0.02 | 0.22 | 0.827 |
| Other | 36 (40.00) | 4.78, (2.7) | 1.77 | **0.08** | -2.5 (1.87) | -0.1 | -1.34 | 0.185 |
| *2010 was chosen because PRISMA was first published in 2009. | | | | | | | | |
