# Supplementary material for: Reporting and methodological quality of systematic reviews underpinning clinical practice guidelines for low back pain: a meta-epidemiological study
Source: Front Pain Res (Lausanne). 2025 Dec 3;6:1704833. doi: 10.3389/fpain.2025.1704833 (PMC12708511; doi:10.3389/fpain.2025.1704833)
Supplement: Supplementary file 5 [file Table5.docx]

| **Supplementary Table 2. Summary of PRISMA Completeness Scores Across the eight included guidelines.** | | | | | | | | | | | |
| --- | --- | --- | --- | --- | --- | --- | --- | --- | --- | --- | --- |
| **PRISMA Item** | **Hawk et al. (n = 5)** | **Manchikati et al. (n = 21)** | **Yabuki et al. (n = 11)** | **Wenger et al.**  **(n= 2)** | **Qaseem et al.**  **(n= 23)** | **Lee et al.**  **(n= 16)** | **Navani et al.**  **(n= 21)** | **Bussieres et al.**  **(n= 11)** | **Mean of all Included Studies (n= 90)** | **Cochrane (n= 22)** | **Non-cochrane (n= 68)** |
|  | Item Scores: M, (SD) | Item Scores: M, (SD) | Item Scores: M, (SD) | Item Scores: M, (SD) | Item Scores: M, (SD) | Item Scores: M, (SD) | Item Scores: M, (SD) | Item Scores: M, (SD) | Item Scores: M, (SD) | Item Scores: M, (SD) | Item Scores: M, (SD) |
| 1. Title: systematic review, meta-analysis, both? | 0.43, (0.51) | 1, (0) | 0.8, (0.42) | 1, (0) | 0.88, (0.34) | 0.64, (0.5) | 0.95, (0.22) | 1, (0) | 0.77, (0.43) | 0.09, (0.29) | 0.99, (0.12) |
| 2. Abstract: structured summary? | 1, (0) | 1, (0) | 1, (0) | 0, (0) | 0, (0) | 1, (0) | 0.67, (0.58) | 1, (0) | 0.75, (0.46) | 0, (0) | 0.75, (0.46) |
| 3. Introduction: rationale for review? | 1, (0) | 1, (0) | 1, (0) | 1, (0) | 1, (0) | 1, (0) | 1, (0) | 1, (0) | 1, (0) | 1, (0) | 1, (0) |
| 4. Introduction: explicit statement of objectives? | 0.76, (0.33) | 0.25, (0.35) | 0.6, (0.39) | 0.52, (0.25) | 0.53, (0.39) | 0.68, (0.4) | 0.45, (0.35) | 0.4, (0.42) | 0.59, (0.36) | 0.93, (0.18) | 0.49, (0.33) |
| 5. Methods: protocol and registration? | 0.54, (0.14) | 1, (0) | 0.7, (0.26) | 0.5, (0) | 0.5, (0.18) | 0.55, (0.27) | 0.52, (0.25) | 0.7, (0.27) | 0.54, (0.21) | 0.5, (0) | 0.56, (0.24) |
| 6. Methods: eligibility criteria? | 1, (0) | 1, (0) | 1, (0) | 0.93, (0.18) | 0.94, (0.17) | 0.95, (0.15) | 0.88, (0.22) | 1, (0) | 0.95, (0.15) | 1, (0) | 0.93, (0.17) |
| 7. Methods: information sources? | 0.78, (0.25) | 0.5, (0) | 0.8, (0.26) | 0.62, (0.22) | 0.69, (0.25) | 0.77, (0.26) | 0.55, (0.22) | 0.7, (0.27) | 0.71, (0.26) | 0.89, (0.21) | 0.65, (0.25) |
| 8. Methods: full search strategy? | 0.8, (0.29) | 0.5, (0) | 0.9, (0.21) | 0.57, (0.33) | 0.75, (0.37) | 0.77, (0.41) | 0.64, (0.42) | 0.6, (0.42) | 0.71, (0.36) | 0.93, (0.23) | 0.64, (0.36) |
| 9. Methods: study selection process? | 1, (0) | 1, (0) | 1, (0) | 1, (0) | 0.94, (0.17) | 0.95, (0.15) | 0.93, (0.18) | 1, (0) | 0.97, (0.13) | 1, (0) | 0.96, (0.14) |
| 10. Methods: data collection process? | 1, (0) | 1, (0) | 1, (0) | 1, (0) | 0.88, (0.34) | 1, (0) | 0.86, (0.32) | 1, (0) | 0.94, (0.22) | 1, (0) | 0.93, (0.25) |
| 11. Methods: data items to be extracted? | 0.67, (0.24) | 0.75, (0.35) | 0.6, (0.21) | 0.74, (0.26) | 0.66, (0.24) | 0.73, (0.26) | 0.76, (0.26) | 0.5, (0) | 0.68, (0.24) | 0.75, (0.26) | 0.66, (0.24) |
| 12. Methods: risk of bias of individual studies? | 0.93, (0.23) | 0.25, (0.35) | 0.8, (0.35) | 1, (0) | 0.94, (0.25) | 0.91, (0.3) | 0.9, (0.26) | 1, (0) | 0.92, (0.25) | 1, (0) | 0.9, (0.28) |
| 13. Methods: summary measures? | 0.98, (0.1) | 0.75, (0.35) | 0.8, (0.42) | 0.67, (0.48) | 0.81, (0.4) | 0.68, (0.46) | 0.71, (0.46) | 0.8, (0.45) | 0.79, (0.4) | 1, (0) | 0.72, (0.44) |
| 14. Methods: synthesis of results? | 1, (0) | 0, (0) | 1, (0) | 1, (0) | 0.92, (0.29) | 1, (0) | 1, (0) | 1, (0) | 0.98, (0.13) | 1, (0) | 0.98, (0.15) |
| 15. Methods: risk of bias across studies? | 0.65, (0.41) | 0, (0) | 0.6, (0.46) | 0.6, (0.37) | 0.5, (0.45) | 0.86, (0.32) | 0.48, (0.33) | 0.4, (0.42) | 0.62, (0.39) | 0.86, (0.23) | 0.54, (0.4) |
| 16. Methods: additional analyses? | 0.84, (0.37) | 1, (0) | 1, (0) | 0.78, (0.44) | 0.93, (0.19) | 0.86, (0.38) | 0.83, (0.39) | 0.75, (0.5) | 0.86, (0.35) | 0.89, (0.32) | 0.83, (0.37) |
| 17. Results: study selection? | 0.78, (0.25) | 1, (0) | 1, (0) | 0.98, (0.11) | 0.94, (0.17) | 1, (0) | 0.9, (0.26) | 1, (0) | 0.91, (0.21) | 0.84, (0.24) | 0.93, (0.19) |
| 18. Results: study characteristics? | 1, (0) | 1, (0) | 0.9, (0.21) | 1, (0) | 1, (0) | 0.95, (0.15) | 0.95, (0.15) | 1, (0) | 0.98, (0.1) | 1, (0) | 0.97, (0.12) |
| 19. Results: risk of bias within studies? | 0.98, (0.1) | 0.75, (0.35) | 0.7, (0.42) | 1, (0) | 0.94, (0.25) | 0.91, (0.3) | 0.86, (0.36) | 1, (0) | 0.91, (0.28) | 1, (0) | 0.88, (0.31) |
| 20. Results: results of individual studies? | 0.83, (0.32) | 0, (0) | 0.75, (0.35) | 0.71, (0.34) | 0.81, (0.31) | 0.77, (0.34) | 0.76, (0.34) | 0.9, (0.22) | 0.79, (0.32) | 0.95, (0.15) | 0.74, (0.34) |
| 21. Results: synthesis of results? | 1, (0) | 0, (0) | 1, (0) | 1, (0) | 0.92, (0.29) | 1, (0) | 1, (0) | 1, (0) | 0.98, (0.13) | 1, (0) | 0.98, (0.15) |
| 22. Results: risk of bias across studies? | 0.91, (0.25) | 0.25, (0.35) | 0.7, (0.42) | 1, (0) | 0.88, (0.34) | 0.91, (0.3) | 0.9, (0.26) | 1, (0) | 0.89, (0.29) | 1, (0) | 0.86, (0.32) |
| 23. Results: results of additional analyses? | 1, (0) | 0, (0) | 1, (0) | 1, (0) | 0.92, (0.29) | 1, (0) | 1, (0) | 1, (0) | 0.98, (0.13) | 1, (0) | 0.98, (0.15) |
| 24. Discussion: summary of evidence? | 0.91, (0.25) | 0.25, (0.35) | 0.7, (0.42) | 1, (0) | 0.88, (0.34) | 0.91, (0.3) | 0.9, (0.26) | 1, (0) | 0.89, (0.29) | 1, (0) | 0.86, (0.32) |
| 25. Discussion: study limitations? | 0.98, (0.1) | 1, (0) | 1, (0) | 0.9, (0.26) | 1, (0) | 0.95, (0.15) | 0.74, (0.44) | 1, (0) | 0.91, (0.27) | 0.98, (0.11) | 0.89, (0.3) |
| 26. Discussion: conclusions? | 1, (0) | 1, (0) | 0.8, (0.42) | 0.64, (0.48) | 0.75, (0.45) | 1, (0) | 0.62, (0.47) | 0.9, (0.22) | 0.83, (0.36) | 0.95, (0.21) | 0.79, (0.39) |
| 27. Funding: funding sources and role of funders? | 0.91, (0.29) | 1, (0) | 0.95, (0.16) | 0.69, (0.46) | 0.84, (0.3) | 0.91, (0.3) | 0.86, (0.36) | 0.8, (0.45) | 0.85, (0.35) | 1, (0) | 0.8, (0.39) |
| **Overall PRISMA completeness %, (SD):** | 87.29, (5.38) | 76, (2.83) | 84.55, (9.73) | 81.4, (5.43) | 82.09, (14.14) | 85.4, (15.95) | 77.87, (15.58) | 84.6, (7.31) | 82.96, (12.23) | 90.58, (2.64) | 80.49, (13.09) |
