# Supplementary material for: Reporting and methodological quality of systematic reviews underpinning clinical practice guidelines for low back pain: a meta-epidemiological study
Source: Front Pain Res (Lausanne). 2025 Dec 3;6:1704833. doi: 10.3389/fpain.2025.1704833 (PMC12708511; doi:10.3389/fpain.2025.1704833)
Supplement: Supplementary file 6 [file Table6.docx]

| **Supplementary Table 3. Summary of AMSTAR completeness scores across the eight included guidelines.** | | | | | | | | | | | |
| --- | --- | --- | --- | --- | --- | --- | --- | --- | --- | --- | --- |
| **AMSTAR-2 Item** | Hawk et al. (n = 5) | Manchikati et al. (n = 21) | Yabuki et al. (n = 11) | Wenger et al.  (n= 2) | Qaseem et al.  (n= 23) | Lee et al.  (n= 16) | Navani et al.  (n= 21) | Bussieres et al.  (n= 11) | Mean of all Included Studies (n= 90) | Cochrane (n= 22) | Non-cochrane (n= 68) |
|  | Item Scores: M, (SD) | Item Scores: M, (SD) | Item Scores: M, (SD) | Item Scores: M, (SD) | Item Scores: M, (SD) | Item Scores: M, (SD) | Item Scores: M, (SD) | Item Scores: M, (SD) | Item Scores: M, (SD) | Item Scores: M, (SD) | Item Scores: M, (SD) |
| 1) Did the research questions and inclusion criteria for the review include the components of PICO? | 1, (0) | 1, (0) | 1, (0) | 1, (0) | 1, (0) | 0.91, (0.3) | 0.9, (0.3) | 1, (0) | 0.97, (0.18) | 1, (0) | 0.96, (0.21) |
| 2) Did the report of the review contain an explicit statement that the review methods were established prior to the conduct of the review and did the report justify any significant deviations from the protocol? | 0.57, (0.17) | 1, (0) | 0.75, (0.26) | 0.5, (0) | 0.5, (0.26) | 0.55, (0.27) | 0.57, (0.18) | 0.7, (0.27) | 0.56, (0.21) | 0.52, (0.11) | 0.57, (0.23) |
| 3) Did the review authors explain their selection of the study designs for inclusion in the review? | 0.91, (0.29) | 1, (0) | 1, (0) | 1, (0) | 0.94, (0.25) | 0.91, (0.3) | 0.95, (0.22) | 1, (0) | 0.94, (0.23) | 1, (0) | 0.93, (0.26) |
| 4) Did the review authors use a comprehensive literature search strategy? | 0.63, (0.22) | 0.5, (0) | 0.65, (0.34) | 0.55, (0.31) | 0.53, (0.29) | 0.59, (0.3) | 0.62, (0.27) | 0.6, (0.22) | 0.59, (0.28) | 0.64, (0.28) | 0.58, (0.28) |
| 5) Did the review authors perform study selection in duplicate? | 1, (0) | 1, (0) | 1, (0) | 1, (0) | 0.88, (0.34) | 1, (0) | 0.86, (0.36) | 1, (0) | 0.94, (0.23) | 1, (0) | 0.93, (0.26) |
| 6) Did the review authors perform data extraction in duplicate? | 1, (0) | 1, (0) | 1, (0) | 1, (0) | 0.88, (0.34) | 1, (0) | 0.86, (0.36) | 1, (0) | 0.94, (0.23) | 1, (0) | 0.93, (0.26) |
| 7) Did the review authors provide a list of excluded studies and justify the exclusions? | 0.72, (0.45) | 0, (0) | 0.25, (0.42) | 0.33, (0.43) | 0.41, (0.46) | 0.45, (0.52) | 0.43, (0.46) | 0.2, (0.45) | 0.46, (0.47) | 0.91, (0.29) | 0.31, (0.42) |
| 8) Did the review authors describe the included studies in adequate detail? | 1, (0) | 1, (0) | 0.95, (0.16) | 1, (0) | 0.97, (0.13) | 0.95, (0.15) | 0.93, (0.18) | 0.9, (0.22) | 0.97, (0.13) | 0.98, (0.11) | 0.96, (0.13) |
| 9) Did the review authors use a satisfactory technique for assessing the risk of bias (RoB) in individual studies that were included in the review? | 0.98, (0.1) | 0.75, (0.35) | 0.8, (0.35) | 0.95, (0.15) | 0.88, (0.29) | 0.86, (0.32) | 0.88, (0.31) | 1, (0) | 0.9, (0.26) | 1, (0) | 0.87, (0.29) |
| 10 ) Did the review authors report on the sources of funding for the studies included in the review? | 0.43, (0.51) | 0.5, (0.71) | 0.3, (0.48) | 0.48, (0.51) | 0.38, (0.5) | 0.45, (0.52) | 0.57, (0.51) | 0.2, (0.45) | 0.42, (0.5) | 0.59, (0.5) | 0.37, (0.49) |
| 11) If meta-analysis was performed did the review authors use appropriate methods for statistical combination of results? | 1, (0) | 0, (0) | 0.83, (0.41) | 1, (0) | 0.83, (0.39) | 1, (0) | 1, (0) | 1, (0) | 0.97, (0.18) | 1, (0) | 0.95, (0.21) |
| 12) If meta-analysis was performed, did the review authors assess the potential impact of RoB in individual studies on the results of the meta-analysis or other evidence synthesis? | 0.95, (0.23) | 0, (0) | 1, (0) | 1, (0) | 0.92, (0.29) | 1, (0) | 0.92, (0.28) | 1, (0) | 0.95, (0.21) | 1, (0) | 0.93, (0.26) |
| 13) Did the review authors account for RoB in individual studies when interpreting/ discussing the results of the review? | 0.96, (0.21) | 1, (0) | 0.9, (0.32) | 1, (0) | 0.88, (0.34) | 0.91, (0.3) | 0.9, (0.3) | 1, (0) | 0.92, (0.27) | 1, (0) | 0.9, (0.31) |
| 14) Did the review authors provide a satisfactory explanation for, and discussion of, any heterogeneity observed in the results of the review? | 0.96, (0.21) | 1, (0) | 0.7, (0.48) | 0.86, (0.36) | 0.75, (0.45) | 0.82, (0.4) | 0.57, (0.51) | 0.8, (0.45) | 0.79, (0.41) | 0.95, (0.21) | 0.74, (0.44) |
| 15) If they performed quantitative synthesis did the review authors carry out an adequate investigation of publication bias (small study bias) and discuss its likely impact on the results of the review? | 0.47, (0.51) | 0, (0) | 0.67, (0.52) | 0.33, (0.49) | 0.46, (0.52) | 0.71, (0.49) | 0.31, (0.48) | 0.33, (0.58) | 0.47, (0.5) | 0.5, (0.51) | 0.45, (0.5) |
| 16) Did the review authors report any potential sources of conflict of interest, including any funding they received for conducting the review? | 0.91, (0.29) | 1, (0) | 1, (0) | 0.81, (0.4) | 1, (0) | 1, (0) | 0.9, (0.3) | 1, (0) | 0.93, (0.25) | 1, (0) | 0.91, (0.29) |
| **AMSTAR Score** | 84.45, (9.51) | 82.69, (8.16) | 79.38, (11.34) | 80.15, (6.1) | 75.93, (19.65) | 80.51, (17.66) | 76.02, (16.27) | 80.38, (11.22) | 79.32, (14.4) | 88.22, (6.35) | 76.43, (15.11) |
