# Supplementary material for: Reporting and methodological quality of systematic reviews underpinning clinical practice guidelines for low back pain: a meta-epidemiological study
Source: Front Pain Res (Lausanne). 2025 Dec 3;6:1704833. doi: 10.3389/fpain.2025.1704833 (PMC12708511; doi:10.3389/fpain.2025.1704833)
Supplement: Supplementary file 7 [file Table7.docx]

| **Supplementary Table 4. Quality of Systematic Reviews Included in CPGs** | | | | | | | | |
| --- | --- | --- | --- | --- | --- | --- | --- | --- |
|  | **Title of SR and/or MA** | **Authors** | **PRISMA Score**** | **AMSTAR 2 Score**** | **AMSTAR 2 Critical Appraisal** | **PMID of SR** | **Link to SR/MA if applicable** | **CPGs** |
| 1 | The effect of antidepressant treatment on chronic back pain: a meta-analysis | Salerno et al. 2002 | 75 | 78.13 | Moderate | 11784215 | <https://pubmed.ncbi.nlm.nih.gov/11784215/> | Noninvasive Treatments for Acute, Subacute, and Chronic Low Back Pain: A Clinical Practice Guideline From the American College of Physicians |
| 2 | Muscle relaxants for non-specific low back pain* | Tulder et al. 2003 | 90.38 | 96.88 | High | 12804507 | <https://pubmed.ncbi.nlm.nih.gov/12804507/> | Noninvasive Treatments for Acute, Subacute, and Chronic Low Back Pain: A Clinical Practice Guideline From the American College of Physicians |
| 3 | Meta-analysis: acupuncture for low back pain | Manheimer et al. 2005 | 90.38 | 90.63 | Moderate | 15838072 | <https://pubmed.ncbi.nlm.nih.gov/15838072/> | Noninvasive Treatments for Acute, Subacute, and Chronic Low Back Pain: A Clinical Practice Guideline From the American College of Physicians |
| 4 | Superficial heat or cold for low back pain* | French et al. 2006 | 89.58 | 96.15 | Moderate | 16437495 | <https://pubmed.ncbi.nlm.nih.gov/16437495/> | Noninvasive Treatments for Acute, Subacute, and Chronic Low Back Pain: A Clinical Practice Guideline From the American College of Physicians |
| 5 | Medial branch neurotomy in management of chronic spinal pain: systematic review of the evidence | Manchikanti et al. 2002 | 29.17 | 34.62 | Low | 16886020 | <https://pubmed.ncbi.nlm.nih.gov/16886020/> | Comprehensive Evidence-Based Guidelines for Facet Joint Interventions in the Management  of Chronic Spinal Pain: American Society of Interventional Pain Physicians (ASIPP) Guidelines |
| 6 | Systematic review of effectiveness and complications of adhesiolysis in the management of chronic spinal pain: an update | Trescot et al. 2007 | 56.82 | 34.62 | Critically low | 17256027 | <https://pubmed.ncbi.nlm.nih.gov/17256027/> | Nonsurgical treatments for patients with radicular pain from lumbosacral disc herniation |
| 7 | Nonpharmacologic therapies for acute and chronic low back pain: a review of the evidence for an American Pain Society/American College of Physicians clinical practice guideline | Chou et al. 2007 | 79.55 | 73.08 | Moderate | 17909210 | <https://pubmed.ncbi.nlm.nih.gov/17909210/> | Nonsurgical treatments for patients with radicular pain from lumbosacral disc herniation  Spinal Manipulative Therapy and Other Conservative Treatments for Low Back Pain: A Guideline From the Canadian Chiropractic Guideline Initiative |
| 8 | Non-steroidal anti-inflammatory drugs for low back pain* | Roelofs et al. 2008 | 86.54 | 81.25 | Moderate | 18253976 | <https://pubmed.ncbi.nlm.nih.gov/18253976/> | Noninvasive Treatments for Acute, Subacute, and Chronic Low Back Pain: A Clinical Practice Guideline From the American College of Physicians |
| 9 | Antidepressants for non-specific low back pain* | Urquhart et al. 2008 | 86.54 | 75 | Moderate | 18253994 | <https://pubmed.ncbi.nlm.nih.gov/18253994/> | Noninvasive Treatments for Acute, Subacute, and Chronic Low Back Pain: A Clinical Practice Guideline From the American College of Physicians |
| 10 | Massage for low-back pain* | Furlan et al. 2008 | 86.54 | 87.5 | High | 18843627 | <https://pubmed.ncbi.nlm.nih.gov/18843627/> | Noninvasive Treatments for Acute, Subacute, and Chronic Low Back Pain: A Clinical Practice Guideline From the American College of Physicians |
| 11 | Conservative management of lumbar disc herniation with associated radiculopathy: a systematic review | Hahne et al. 2010 | 86.54 | 75 | Moderate | 20421859 | <https://pubmed.ncbi.nlm.nih.gov/20421859/> | Nonsurgical treatments for patients with radicular pain from lumbosacral disc herniation  Responsible, Safe, and Effective Use of Biologics in the Management of Low Back Pain: American Society of Interventional Pain Physicians (ASIPP) Guidelines |
| 12 | Behavioural treatment for chronic low-back pain* | Henschke et al. 2010 | 88.46 | 81.25 | Moderate | 20614428 | <https://pubmed.ncbi.nlm.nih.gov/20614428/> | Noninvasive Treatments for Acute, Subacute, and Chronic Low Back Pain: A Clinical Practice Guideline From the American College of Physicians |
| 13 | A systematic review on the effectiveness of physical and rehabilitation interventions for chronic non-specific low back pain | Middelkoop et al. 2010 | 88 | 81.25 | Moderate | 20640863 | <https://pubmed.ncbi.nlm.nih.gov/20640863/> | Noninvasive Treatments for Acute, Subacute, and Chronic Low Back Pain: A Clinical Practice Guideline From the American College of Physicians |
| 14 | Spinal manipulative therapy for chronic low-back pain* | Rubinstein et al. 2011 | 92.31 | 93.75 | High | 21328304 | <https://pubmed.ncbi.nlm.nih.gov/21328304/> | Noninvasive Treatments for Acute, Subacute, and Chronic Low Back Pain: A Clinical Practice Guideline From the American College of Physicians |
| 15 | Efficacy and tolerance of systemic steroids in sciatica: a systematic review and meta-analysis | Roncoroni et al. 2011 | 84.62 | 84.38 | Moderate | 21525139 | <https://pubmed.ncbi.nlm.nih.gov/21525139/> | Nonsurgical treatments for patients with radicular pain from lumbosacral disc herniation |
| 16 | Spinal manipulative therapy for chronic low-back pain: an update of a Cochrane review* | Rubinstein et al. 2011 | 88.46 | 93.75 | High | 21593658 | <https://pubmed.ncbi.nlm.nih.gov/21593658/> | Spinal Manipulative Therapy and Other Conservative Treatments for Low Back Pain: A Guideline From the Canadian Chiropractic Guideline Initiative |
| 17 | A systematic review and meta-analysis of efficacy, cost-effectiveness, and safety of selected complementary and alternative medicine for neck and low-back pain | Furlan et al. 2011 | 92.31 | 78.13 | Moderate | 22203884 | <https://pubmed.ncbi.nlm.nih.gov/22203884/> | Comprehensive Evidence-Based Guidelines for Facet Joint Interventions in the Management of Chronic Spinal Pain: American Society of Interventional Pain Physicians (ASIPP) Guidelines  Spinal Manipulative Therapy and Other Conservative Treatments for Low Back Pain: A Guideline From the Canadian Chiropractic Guideline Initiative |
| 18 | Antineuropathic and antinociceptive drugs combination in patients with chronic low back pain: a systematic review | Romano et al. 2012 | 40.91 | 30.77 | Critically low | 22619711 | <https://pubmed.ncbi.nlm.nih.gov/22619711/> | Evidence-Based Recommendations on the Pharmacological Management of Osteoarthritis and Chronic Low Back Pain: An Asian Consensus |
| 19 | Spinal manipulative therapy for acute low-back pain* | Rubinstein et al. 2012 | 94.23 | 96.88 | High | 22972127 | <https://pubmed.ncbi.nlm.nih.gov/22972127/> | Noninvasive Treatments for Acute, Subacute, and Chronic Low Back Pain: A Clinical Practice Guideline From the American College of Physicians |
| 20 | A systematic review and meta-analysis of yoga for low back pain | Cramer et al. 2013 | 86.54 | 87.5 | High | 23246998 | <https://pubmed.ncbi.nlm.nih.gov/23246998/> | Noninvasive Treatments for Acute, Subacute, and Chronic Low Back Pain: A Clinical Practice Guideline From the American College of Physicians |
| 21 | Acupuncture for acute low back pain: a systematic review | Lee et al. 2013 | 86.54 | 75 | Moderate | 23269281 | <https://pubmed.ncbi.nlm.nih.gov/23269281/> | Noninvasive Treatments for Acute, Subacute, and Chronic Low Back Pain: A Clinical Practice Guideline From the American College of Physicians |
| 22 | Motor control exercises reduces pain and disability in chronic and recurrent low back pain: a meta-analysis | Bystrom et al. 2013 | 85.42 | 75 | Moderate | 23492976 | <https://pubmed.ncbi.nlm.nih.gov/23492976/> | Noninvasive Treatments for Acute, Subacute, and Chronic Low Back Pain: A Clinical Practice Guideline From the American College of Physicians |
| 23 | Serious Adverse Events and Spinal Manipulative Therapy of the Low Back Region: A Systematic Review of Cases | Hebert et al. 2013 | 65.91 | 53.85 | Critically low | 23787298 | <https://pubmed.ncbi.nlm.nih.gov/23787298/> | Spinal Manipulative Therapy and Other Conservative Treatments for Low Back Pain: A Guideline From the Canadian Chiropractic Guideline Initiative |
| 24 | Traction for low-back pain with or without sciatica* | Weger et al. 2013 | 86.54 | 81.25 | Moderate | 23959683 | <https://pubmed.ncbi.nlm.nih.gov/23959683/> | Noninvasive Treatments for Acute, Subacute, and Chronic Low Back Pain: A Clinical Practice Guideline From the American College of Physicians |
| 25 | Opioids compared to placebo or other treatments for chronic low-back pain* | Chaparro et al. 2013 | 90.38 | 93.75 | High | 23983011 | <https://pubmed.ncbi.nlm.nih.gov/23983011/> | Evidence-Based Recommendations on the Pharmacological Management of Osteoarthritis and Chronic Low Back Pain: An Asian Consensus  Noninvasive Treatments for Acute, Subacute, and Chronic Low Back Pain: A Clinical Practice Guideline From the American College of Physicians |
| 26 | Effectiveness of acupuncture for nonspecific chronic low back pain: a systematic review and meta-analysis | Lam et al. 2013 | 90.38 | 56.25 | Low | 24026151 | <https://pubmed.ncbi.nlm.nih.gov/24026151/> | Noninvasive Treatments for Acute, Subacute, and Chronic Low Back Pain: A Clinical Practice Guideline From the American College of Physicians |
| 27 | Drug therapy for the treatment of chronic nonspecific low back pain: systematic review and meta-analysis | Chung et al. 2013 | 86.54 | 75 | Moderate | 24284847 | <https://pubmed.ncbi.nlm.nih.gov/24284847/> | Evidence-Based Recommendations on the Pharmacological Management of Osteoarthritis and Chronic Low Back Pain: An Asian Consensus |
| 28 | Do Epidural Injections Provide Short- and Long-term Relief for Lumbar Disc Herniation? A Systematic Review | Manchikanti et al. 2015 | 79.55 | 82.14 | Low | 24515404 | <https://pubmed.ncbi.nlm.nih.gov/24515404/> | Nonsurgical treatments for patients with radicular pain from lumbosacral disc herniation  Responsible, Safe, and Effective Use of Biologics in the Management of Low Back Pain: American Society of Interventional Pain Physicians (ASIPP) Guidelines |
| 29 | Therapeutic ultrasound for chronic low-back pain* | Ebadi et al. 2014 | 91.67 | 90.63 | High | 24627326 | <https://pubmed.ncbi.nlm.nih.gov/24627326/> | Noninvasive Treatments for Acute, Subacute, and Chronic Low Back Pain: A Clinical Practice Guideline From the American College of Physicians |
| 30 | Clinical effectiveness of manual therapy for the management of musculoskeletal and non-musculoskeletal conditions: systematic review and update of UK evidence report | Clar et al. 2014 | 75 | 76.92 | Moderate | 24679336 | <https://pubmed.ncbi.nlm.nih.gov/24679336/> | Best Practices for Chiropractic Management of Patients with Chronic Musculoskeletal Pain: A Clinical Practice Guideline |
| 31 | Radiofrequency denervation for facet joint low back pain: a systematic review | Poetscher et al. 2014 | 96.15 | 90.63 | High | 24732848 | <https://pubmed.ncbi.nlm.nih.gov/24732848/> | Comprehensive Evidence-Based Guidelines for Facet Joint Interventions in the Management of Chronic Spinal Pain: American Society of Interventional Pain Physicians (ASIPP) Guidelines |
| 32 | Epidural steroid injections for radicular lumbosacral pain: a systematic review | Shamliyan et al. 2014 | 86 | 100 | Moderate | 24787344 | <https://pubmed.ncbi.nlm.nih.gov/24787344/> | Nonsurgical treatments for patients with radicular pain from lumbosacral disc herniation |
| 33 | Sling exercise for chronic low back pain: a systematic review and meta-analysis | Yue et al. 2014 | 94.23 | 87.5 | High | 24919119 | <https://pubmed.ncbi.nlm.nih.gov/24919119/> | Nonsurgical treatments for patients with radicular pain from lumbosacral disc herniation |
| 34 | The effectiveness of Pilates exercise in people with chronic low back pain: a systematic review | Wells et al. 2014 | 84.09 | 80.77 | Moderate | 24984069 | <https://pubmed.ncbi.nlm.nih.gov/24984069/> | Noninvasive Treatments for Acute, Subacute, and Chronic Low Back Pain: A Clinical Practice Guideline From the American College of Physicians |
| 35 | Transforaminal versus interlaminar approaches to epidural steroid injections: a systematic review of comparative studies for lumbosacral radicular pain | Chang-Chien et al. 2014 | 89.58 | 87.5 | High | 25054401 | <https://pubmed.ncbi.nlm.nih.gov/25054387/> | Nonsurgical treatments for patients with radicular pain from lumbosacral disc herniation |
| 36 | Radiofrequency ablation for chronic low back pain: a systematic review of randomized controlled trials | Leggett et al. 2014 | 81.82 | 76.92 | Moderate | 25068973 | <https://pubmed.ncbi.nlm.nih.gov/25068973/> | Comprehensive Evidence-Based Guidelines for Facet Joint Interventions in the Management of Chronic Spinal Pain: American Society of Interventional Pain Physicians (ASIPP) Guidelines |
| 37 | Multidisciplinary biopsychosocial rehabilitation for chronic low back pain* | Kamper et al. 2014 | 92.31 | 87.5 | High | 25180773 | <https://pubmed.ncbi.nlm.nih.gov/25180773/> | Noninvasive Treatments for Acute, Subacute, and Chronic Low Back Pain: A Clinical Practice Guideline From the American College of Physicians |
| 38 | Epidural injections in prevention of surgery for spinal pain: systematic review and meta-analysis of randomized controlled trials | Bicket et al. 2014 | 92.31 | 84.38 | High | 25463400 | <https://pubmed.ncbi.nlm.nih.gov/25463400/> | Nonsurgical treatments for patients with radicular pain from lumbosacral disc herniation |
| 39 | Steroid for epidural injection in spinal stenosis: a systematic review and meta-analysis | Liu et al. 2015 | 82.69 | 75 | Moderate | 25678775 | <https://pubmed.ncbi.nlm.nih.gov/25678775/> | Responsible, Safe, and Effective Use of Biologics in the Management of Low Back Pain: American Society of Interventional Pain Physicians (ASIPP) Guidelines |
| 40 | Multidisciplinary biopsychosocial rehabilitation for chronic low back pain: Cochrane systematic review and meta-analysis* | Kamper et al. 2015 | 92.31 | 78.13 | Moderate | 25694111 | <https://pubmed.ncbi.nlm.nih.gov/25694111/> | Best Practices for Chiropractic Management of Patients with Chronic Musculoskeletal Pain: A Clinical Practice Guideline |
| 41 | Efficacy of epidural injections in the treatment of lumbar central spinal stenosis: a systematic review | Manchikanti et al. 2015 | 75 | 80.77 | Low | 25789241 | <https://pubmed.ncbi.nlm.nih.gov/25789241/> | Responsible, Safe, and Effective Use of Biologics in the Management of Low Back Pain: American Society of Interventional Pain Physicians (ASIPP) Guidelines |
| 42 | Are non-steroidal anti-inflammatory drugs effective for the management of neck pain and associated disorders, whiplash-associated disorders, or non-specific low back pain? A systematic review of systematic reviews by the Ontario Protocol for Traffic Injury Management (OPTIMa) Collaboration | Wong et al. 2015 | 91.3 | 88.46 | Moderate | 25827308 | <https://pubmed.ncbi.nlm.nih.gov/25827308/> | Evidence-Based Recommendations on the Pharmacological Management of Osteoarthritis and Chronic Low Back Pain: An Asian Consensus |
| 43 | Efficacy and safety of paracetamol for spinal pain and osteoarthritis: systematic review and meta-analysis of randomised placebo controlled trials | Machado et al. 2015 | 100 | 87.5 | Moderate | 25828856 | <https://pubmed.ncbi.nlm.nih.gov/25828856/> | Evidence-Based Recommendations on the Pharmacological Management of Osteoarthritis and Chronic Low Back Pain: An Asian Consensus |
| 44 | What is the Role of Epidural Injections in the Treatment of Lumbar Discogenic Pain: A Systematic Review of Comparative Analysis with Fusion | Manchikanti et al. 2015 | 72.73 | 73.08 | Moderate | 25852828 | <https://pubmed.ncbi.nlm.nih.gov/25852828/> | Responsible, Safe, and Effective Use of Biologics in the Management of Low Back Pain: American Society of Interventional Pain Physicians (ASIPP) Guidelines |
| 45 | Comparison of the efficacy of saline, local anesthetics, and steroids in epidural and facet joint injections for the management of spinal pain: A systematic review of randomized controlled trials | Manchikanti et al. 2015 | 72.73 | 69.23 | Moderate | 26005584 | <https://pubmed.ncbi.nlm.nih.gov/26005584/> | Comprehensive Evidence-Based Guidelines for Facet Joint Interventions in the Management  of Chronic Spinal Pain: American Society of Interventional Pain Physicians (ASIPP) Guidelines  Responsible, Safe, and Effective Use of Biologics in the Management of Low Back Pain: American Society of Interventional Pain Physicians (ASIPP) Guidelines |
| 46 | Tapentadol for chronic musculoskeletal pain in adults* | Santos et al 2015 | 94.23 | 87.5 | High | 26017279 | <https://pubmed.ncbi.nlm.nih.gov/26017279/> | Evidence-Based Recommendations on the Pharmacological Management of Osteoarthritis and Chronic Low Back Pain: An Asian Consensus |
| 47 | Epidural Injection With or Without Steroid in Managing Chronic Low-Back and Lower Extremity Pain: A Meta-Analysis of 10 Randomized Controlled Trials | Zhai et al. 2017 | 81.25 | 78.13 | Moderate | 26035031 | <https://pubmed.ncbi.nlm.nih.gov/26035031/> | Responsible, Safe, and Effective Use of Biologics in the Management of Low Back Pain: American Society of Interventional Pain Physicians (ASIPP) Guidelines |
| 48 | A Systematic Review and Best Evidence Synthesis of the Effectiveness of Therapeutic Facet Joint Interventions in Managing Chronic Spinal Pain | Manchikanti et al. 2015 | 79.55 | 76.92 | Moderate | 26218948 | <https://pubmed.ncbi.nlm.nih.gov/26218948/> | Comprehensive Evidence-Based Guidelines for Facet Joint Interventions in the Management of Chronic Spinal Pain: American Society of Interventional Pain Physicians (ASIPP) Guidelines  Responsible, Safe, and Effective Use of Biologics in the Management of Low Back Pain: American Society of Interventional Pain Physicians (ASIPP) Guidelines |
| 49 | Epidural Corticosteroid Injections for Radiculopathy and Spinal Stenosis: A Systematic Review and Meta-analysis | Chou et al. 2015 | 78.85 | 81.25 | Moderate | 26302454 | <https://pubmed.ncbi.nlm.nih.gov/26302454/> | Comprehensive Evidence-Based Guidelines for Facet Joint Interventions in the Management  of Chronic Spinal Pain: American Society of Interventional Pain Physicians (ASIPP) Guidelines  Responsible, Safe, and Effective Use of Biologics in the Management of Low Back Pain: American Society of Interventional Pain Physicians (ASIPP) Guidelines |
| 50 | Epidural injection with or without steroid in managing chronic low back and lower extremity pain: ameta-analysis of ten randomized controlled trials | Zhai et al. 2015 | 83.33 | 81.25 | Moderate | 26309483 | <https://pubmed.ncbi.nlm.nih.gov/26309483/> | Nonsurgical treatments for patients with radicular pain from lumbosacral disc herniation  Responsible, Safe, and Effective Use of Biologics in the Management of Low Back Pain: American Society of Interventional Pain Physicians (ASIPP) Guidelines |
| 51 | Epidural injections with or without steroids in managing chronic low back pain secondary to lumbar spinal stenosis: a meta-analysis of 13 randomized controlled trials | Meng et al. 2015 | 85.42 | 78.13 | Moderate | 26316704 | <https://pubmed.ncbi.nlm.nih.gov/26316704/> | Responsible, Safe, and Effective Use of Biologics in the Management of Low Back Pain: American Society of Interventional Pain Physicians (ASIPP) Guidelines |
| 52 | Systematic Review of the Diagnostic Accuracy and Therapeutic Effectiveness of Sacroiliac Joint Interventions | Simopoulos et al. 2015 | 75 | 96.15 | Moderate | 26431129 | <https://pubmed.ncbi.nlm.nih.gov/26431129/> | Comprehensive Evidence-Based Guidelines for Facet Joint Interventions in the Management of Chronic Spinal Pain: American Society of Interventional Pain Physicians (ASIPP) Guidelines  Responsible, Safe, and Effective Use of Biologics in the Management of Low Back Pain: American Society of Interventional Pain Physicians (ASIPP) Guidelines |
| 53 | Radiofrequency denervation for chronic low back pain* | Mass et al. 2015 | 88.46 | 84.38 | Moderate | 26495910 | <https://pubmed.ncbi.nlm.nih.gov/26495910/> | Comprehensive Evidence-Based Guidelines for Facet Joint Interventions in the Management of Chronic Spinal Pain: American Society of Interventional Pain Physicians (ASIPP) Guidelines |
| 54 | Effectiveness of Spinal Cord Stimulation in Chronic Spinal Pain: A Systematic Review | Grider et al. 2016 | 72.73 | 92.31 | Moderate | 26752493 | <https://pubmed.ncbi.nlm.nih.gov/26752493/> | Responsible, Safe, and Effective Use of Biologics in the Management of Low Back Pain: American Society of Interventional Pain Physicians (ASIPP) Guidelines |
| 55 | Prevention of Low Back Pain: A Systematic Review and Meta-analysis | Steffens et al. 2016 | 79.17 | 68.75 | Low | 26752509 | <https://pubmed.ncbi.nlm.nih.gov/26752509/> | Nonsurgical treatments for patients with radicular pain from lumbosacral disc herniation  Spinal Manipulative Therapy and Other Conservative Treatments for Low Back Pain: A Guideline From the Canadian Chiropractic Guideline Initiative |
| 56 | Percutaneous and Endoscopic Adhesiolysis in Managing Low Back and Lower Extremity Pain: A Systematic Review and Meta-analysis | Helm et al. 2016 | 83.33 | 87.5 | High | 26815254 | <https://pubmed.ncbi.nlm.nih.gov/26815254/> | Responsible, Safe, and Effective Use of Biologics in the Management of Low Back Pain: American Society of Interventional Pain Physicians (ASIPP) Guidelines |
| 57 | Non-steroidal anti-inflammatory drugs for chronic low back pain* | Enthoven et al. 2016 | 92.31 | 90.63 | High | 26863524 | <https://pubmed.ncbi.nlm.nih.gov/26863524/> | Evidence-Based Recommendations on the Pharmacological Management of Osteoarthritis and Chronic Low Back Pain: An Asian Consensus |
| 58 | Cell-Based Therapies for Lumbar Discogenic Low Back Pain: Systematic Review and Single-Arm Meta-analysis | Wu et al. 2018 | 86.54 | 78.13 | Low | 26953666 | <https://pubmed.ncbi.nlm.nih.gov/26953666/> | Responsible, Safe, and Effective Use of Biologics in the Management of Low Back Pain: American Society of Interventional Pain Physicians (ASIPP) Guidelines |
| 59 | Epidural Injections for Lumbar Radiculopathy and Spinal Stenosis: A Comparative Systematic Review and Meta-Analysis | Manchikanti et al. 2016 | 85.42 | 81.25 | Moderate | 27008296 | <https://pubmed.ncbi.nlm.nih.gov/27008296/> | Comprehensive Evidence-Based Guidelines for Facet Joint Interventions in the Management  of Chronic Spinal Pain: American Society of Interventional Pain Physicians (ASIPP) Guidelines  Responsible, Safe, and Effective Use of Biologics in the Management of Low Back Pain: American Society of Interventional Pain Physicians (ASIPP) Guidelines |
| 60 | Topical NSAIDs for chronic musculoskeletal pain in adults* | Derry et al. 2016 | 92.31 | 90.63 | High | 27103611 | <https://pubmed.ncbi.nlm.nih.gov/27103611/> | Evidence-Based Recommendations on the Pharmacological Management of Osteoarthritis and Chronic Low Back Pain: An Asian Consensus |
| 61 | Efficacy, Tolerability, and Dose-Dependent Effects of Opioid Analgesics for Low Back Pain: A Systematic Review and Meta-analysis | Shaheed et al. 2016 | 92.31 | 87.5 | High | 27213267 | <https://pubmed.ncbi.nlm.nih.gov/27213267/> | Evidence-Based Recommendations on the Pharmacological Management of Osteoarthritis and Chronic Low Back Pain: An Asian Consensus |
| 62 | Effectiveness and Economic Evaluation of Chiropractic Care for the Treatment of Low Back Pain: A Systematic Review of Pragmatic Studies | Blanchette et al. 2016 | 95.83 | 81.25 | Moderate | 27487116 | <https://pubmed.ncbi.nlm.nih.gov/27487116/> | Spinal Manipulative Therapy and Other Conservative Treatments for Low Back Pain: A Guideline From the Canadian Chiropractic Guideline Initiative |
| 63 | Role of Epidural Injections to Prevent Surgical Intervention in Patients with Chronic Sciatica: A Systematic Review and Meta-Analysis | Bhatti et al. 2016 | 41.67 | 25 | Critically low | 27625909 | <https://pubmed.ncbi.nlm.nih.gov/27625909/> | Nonsurgical treatments for patients with radicular pain from lumbosacral disc herniation |
| 64 | Clinical practice guidelines for the noninvasive management of low back pain: A systematic review by the Ontario Protocol for Traffic Injury Management (OPTIMa) Collaboration | Wong et al. 2016 | 79.55 | 80.77 | Moderate | 27712027 | <https://pubmed.ncbi.nlm.nih.gov/27712027/> | Spinal Manipulative Therapy and Other Conservative Treatments for Low Back Pain: A Guideline From the Canadian Chiropractic Guideline Initiative |
| 65 | Non-steroidal anti-inflammatory drugs for sciatica* | Rasmussen-Barr et al. 2016 | 94.23 | 93.75 | High | 27743405 | <https://pubmed.ncbi.nlm.nih.gov/27743405/> | Nonsurgical treatments for patients with radicular pain from lumbosacral disc herniation |
| 66 | Physical activity and exercise for chronic pain in adults: an overview of Cochrane Reviews* | Geneen et al. 2017 | 90.91 | 88.46 | Low | 28087891 | <https://pubmed.ncbi.nlm.nih.gov/28087891/> | Spinal Manipulative Therapy and Other Conservative Treatments for Low Back Pain: A Guideline From the Canadian Chiropractic Guideline Initiative |
| 67 | Systemic Pharmacologic Therapies for Low Back Pain: A Systematic Review for an American College of Physicians Clinical Practice Guideline | Chou et al. 2017 | 74 | 88.46 | Moderate | 28192790 | <https://pubmed.ncbi.nlm.nih.gov/28192790/> | JAMA Clinical Guidelines Synopsis: Treatment of Low Back Pain  Noninvasive Treatments for Acute, Subacute, and Chronic Low Back Pain: A Clinical Practice Guideline From the American College of Physicians |
| 68 | Nonpharmacologic Therapies for Low Back Pain: A Systematic Review for an American College of Physicians Clinical Practice Guideline | Chou et al. 2017 | 78 | 76.92 | Moderate | 28192793 | <https://pubmed.ncbi.nlm.nih.gov/28192793/> | JAMA Clinical Guidelines Synopsis Treatment of Low Back Pain  Noninvasive Treatments for Acute, Subacute, and Chronic Low Back Pain: A Clinical Practice Guideline From the American College of Physicians  Spinal Manipulative Therapy and Other Conservative Treatments for Low Back Pain: A Guideline From the Canadian Chiropractic Guideline Initiative |
| 69 | Amitriptyline for musculoskeletal complaints: a systematic review | Driest et al. 2017 | 79.55 | 76.92 | Moderate | 28334783 | <https://pubmed.ncbi.nlm.nih.gov/28334783/> | Evidence-Based Recommendations on the Pharmacological Management of Osteoarthritis and Chronic Low Back Pain: An Asian Consensus |
| 70 | Association of Spinal Manipulative Therapy With Clinical Benefit and Harm for Acute Low Back Pain: Systematic Review and Meta-analysis | Paige et al. 2017 | 96.15 | 87.5 | Moderate | 28399251 | <https://pubmed.ncbi.nlm.nih.gov/28399251/> | Spinal Manipulative Therapy and Other Conservative Treatments for Low Back Pain: A Guideline From the Canadian Chiropractic Guideline Initiative |
| 71 | The efficacy of conventional radiofrequency denervation in patients with chronic low back pain originating from the facet joints: a meta-analysis of randomized controlled trials | Lee et al. 2017 | 73.08 | 56.25 | Low | 28576500 | <https://pubmed.ncbi.nlm.nih.gov/28576500/> | Comprehensive Evidence-Based Guidelines for Facet Joint Interventions in the Management of Chronic Spinal Pain: American Society of Interventional Pain Physicians (ASIPP) Guidelines |
| 72 | Exercise for the prevention of low back and pelvic girdle pain in pregnancy: A meta-analysis of randomized controlled trials | Shiri et al. 2017 | 79.17 | 84.38 | Moderate | 28869318 | <https://pubmed.ncbi.nlm.nih.gov/28869318/> | Spinal Manipulative Therapy and Other Conservative Treatments for Low Back Pain: A Guideline From the Canadian Chiropractic Guideline Initiative |
| 73 | A Systematic Review of Mesenchymal Stem Cells in Spinal Cord Injury, Intervertebral Disc Repair and Spinal Fusion | Khan et al. 2018 | 90.38 | 81.25 | Moderate | 28891440 | <https://pubmed.ncbi.nlm.nih.gov/28891440/> | Responsible, Safe, and Effective Use of Biologics in the Management of Low Back Pain: American Society of Interventional Pain Physicians (ASIPP) Guidelines |
| 74 | The effectiveness of walking versus exercise on pain and function in chronic low back pain: a systematic review and meta-analysis of randomized trials | Vanti et al. 2017 | 95.83 | 81.25 | Moderate | 29207885 | <https://pubmed.ncbi.nlm.nih.gov/29207885/> | Nonsurgical treatments for patients with radicular pain from lumbosacral disc herniation |
| 75 | Fusion or Not for Degenerative Lumbar Spinal Stenosis: A Meta-Analysis and Systematic Review | Shen et al. 2018 | 78.85 | 75 | Moderate | 29357326 | <https://pubmed.ncbi.nlm.nih.gov/29357326/> | Comprehensive Evidence-Based Guidelines for Facet Joint Interventions in the Management  of Chronic Spinal Pain: American Society of Interventional Pain Physicians (ASIPP) Guidelines  Responsible, Safe, and Effective Use of Biologics in the Management of Low Back Pain: American Society of Interventional Pain Physicians (ASIPP) Guidelines |
| 76 | Literature Review and Meta-Analysis of Transcutaneous Electrical Nerve Stimulation in Treating Chronic Back Pain | Wu et al. 2018 | 86.54 | 75 | Moderate | 29394211 | <https://pubmed.ncbi.nlm.nih.gov/29394211/> | Best Practices for Chiropractic Management of Patients with Chronic Musculoskeletal Pain: A Clinical Practice Guideline |
| 77 | Transcutaneous electrical nerve stimulation and interferential current demonstrate similar effects in relieving acute and chronic pain: a systematic review with meta-analysis | Almeida et al. 2018 | 79.17 | 71.88 | Moderate | 29426587 | <https://pubmed.ncbi.nlm.nih.gov/29426587/> | Best Practices for Chiropractic Management of Patients with Chronic Musculoskeletal Pain: A Clinical Practice Guideline |
| 78 | Comparison of clinical efficacy of transforaminal and caudal epidural steroid injection in lumbar and lumbosacral disc herniation: A systematic review and meta-analysis | Lee et al. 2018 | 85.42 | 75 | Moderate | 30030083 | <https://pubmed.ncbi.nlm.nih.gov/30030083/> | Nonsurgical treatments for patients with radicular pain from lumbosacral disc herniation |
| 79 | Noninvasive Nonpharmacological Treatment for Chronic Pain: A Systematic Review [Internet] | Skelly et al. 2018 | 98.15 | 93.75 | High | 30179389 | <https://pubmed.ncbi.nlm.nih.gov/30179389/> | Comprehensive Evidence-Based Guidelines for Facet Joint Interventions in the Management of Chronic Spinal Pain: American Society of Interventional Pain Physicians (ASIPP) Guidelines |
| 80 | Comparison of Clinical Efficacy Between Transforaminal and Interlaminar Epidural Injections in Lumbosacral Disc Herniation: A Systematic Review and Meta-Analysis* | Lee et al. 2018 | 84.62 | 81.25 | Moderate | 30282389 | <https://pubmed.ncbi.nlm.nih.gov/30282389/> | Comprehensive Evidence-Based Guidelines for Facet Joint Interventions in the Management  of Chronic Spinal Pain: American Society of Interventional Pain Physicians (ASIPP) Guidelines  Nonsurgical treatments for patients with radicular pain from lumbosacral disc herniation  Responsible, Safe, and Effective Use of Biologics in the Management of Low Back Pain: American Society of Interventional Pain Physicians (ASIPP) Guidelines |
| 81 | Comparison of Clinical Efficacy of Epidural Injection With or Without Steroid in Lumbosacral Disc Herniation: A Systematic Review and Meta-analysis | Lee et al. 2018 | 88.46 | 81.25 | Moderate | 30282390 | <https://pubmed.ncbi.nlm.nih.gov/30282390/> | Comprehensive Evidence-Based Guidelines for Facet Joint Interventions in the Management  of Chronic Spinal Pain: American Society of Interventional Pain Physicians (ASIPP) Guidelines  Responsible, Safe, and Effective Use of Biologics in the Management of Low Back Pain: American Society of Interventional Pain Physicians (ASIPP) Guidelines |
| 82 | Do Regenerative Medicine Therapies Provide Long-Term Relief in Chronic Low Back Pain: A Systematic Review and Metaanalysis | Sanapati et al. 2018 | 86.54 | 78.13 | Moderate | 30508983 | <https://pubmed.ncbi.nlm.nih.gov/30508983/> | Responsible, Safe, and Effective Use of Biologics in the Management of Low Back Pain: American Society of Interventional Pain Physicians (ASIPP) Guidelines |
| 83 | Sacroiliac Joint Fusion Methodology - Minimally Invasive Compared to Screw-Type Surgeries: A Systematic Review and Meta-Analysis | Tran et al. 2019 | 67.31 | 46.88 | Low | 30700066 | <https://pubmed.ncbi.nlm.nih.gov/30700066/> | Comprehensive Evidence-Based Guidelines for Facet Joint Interventions in the Management of Chronic Spinal Pain: American Society of Interventional Pain Physicians (ASIPP) Guidelines |
| 84 | Effectiveness of Percutaneous Adhesiolysis in Post Lumbar Surgery Syndrome: A Systematic Analysis of Findings of Systematic Reviews | Manchikanti et al. 2019 | 59.09 | 73.08 | Low | 31337160 | <https://pubmed.ncbi.nlm.nih.gov/31337160/> | Comprehensive Evidence-Based Guidelines for Facet Joint Interventions in the Management of Chronic Spinal Pain: American Society of Interventional Pain Physicians (ASIPP) Guidelines |
| 85 | Effectiveness of Percutaneous Adhesiolysis in Managing Chronic Central Lumbar Spinal Stenosis: A Systematic Review and Meta-Analysis | Manchikanti et al. 2019 | 85.42 | 84.38 | High | 31775400 | <https://pubmed.ncbi.nlm.nih.gov/31775400/> | Comprehensive Evidence-Based Guidelines for Facet Joint Interventions in the Management of Chronic Spinal Pain: American Society of Interventional Pain Physicians (ASIPP) Guidelines |
| 86 | A methodological quality assessment of systematic reviews and meta-analyses of antidepressants effect on low back pain using updated AMSTAR | Panahi et al. 2020 | 79.55 | 76.92 | Moderate | 31973739 | <https://pubmed.ncbi.nlm.nih.gov/31973739/> | Evidence-Based Recommendations on the Pharmacological Management of Osteoarthritis and Chronic Low Back Pain: An Asian Consensus |
| 87 | Systematic Review of the Effectiveness of Lumbar Medial Branch Thermal Radiofrequency Neurotomy, Stratified for Diagnostic Methods and Procedural Technique | Schneider et al. 2020 | 56.82 | 53.85 | Moderate | 32040149 | <https://pubmed.ncbi.nlm.nih.gov/32040149/> | Comprehensive Evidence-Based Guidelines for Facet Joint Interventions in the Management of Chronic Spinal Pain: American Society of Interventional Pain Physicians (ASIPP) Guidelines |
| 88 | Does Epidural Bupivacaine with or Without Steroids Provide Long-Term Relief? A Systematic Review and Meta-analysis | Manchikanti et al. 2020 | 74 | 81.25 | Moderate | 32335757 | <https://pubmed.ncbi.nlm.nih.gov/32335757/> | Comprehensive Evidence-Based Guidelines for Facet Joint Interventions in the Management of Chronic Spinal Pain: American Society of Interventional Pain Physicians (ASIPP) Guidelines |
| 89 | Noninvasive Nonpharmacological Treatment for Chronic Pain: A Systematic Review Update | Skelly et al. 2020 | 90 | 100 | Moderate | 32338846 | <https://pubmed.ncbi.nlm.nih.gov/32338846/> | Best Practices for Chiropractic Management of Patients with Chronic Musculoskeletal Pain: A Clinical Practice Guideline  Comprehensive Evidence-Based Guidelines for Facet Joint Interventions in the Management of Chronic Spinal Pain: American Society of Interventional Pain Physicians (ASIPP) Guidelines |
| 90 | Therapeutic ultrasound for chronic low back pain* | Ebadi et al. 2020 | 93.75 | 90.63 | High | 32623724 | <https://pubmed.ncbi.nlm.nih.gov/32623724/> | Noninvasive Treatments for Acute, Subacute, and Chronic Low Back Pain: A Clinical Practice Guideline From the American College of Physicians |
| *Denotes a Cochrane systematic review  **PRISMA and AMSTAR-2 scores are proportion of criteria met | | | | | | | | |
