# Supplementary material for: Reporting and methodological quality of systematic reviews underpinning clinical practice guidelines for low back pain: a meta-epidemiological study
Source: Front Pain Res (Lausanne). 2025 Dec 3;6:1704833. doi: 10.3389/fpain.2025.1704833 (PMC12708511; doi:10.3389/fpain.2025.1704833)
Supplement: Supplementary file 8 [file Table8.docx]

| **Supplementary Table 5. Systematic Reviews Analyzed** | | |  |  |  |  |  |
| --- | --- | --- | --- | --- | --- | --- | --- |
|  | 0 | **Title of SR and/or MA** | **Authors** | **PMID of SR** | **Link to SR/MA if applicable** | **CPGs** |  |
| 1 | 1 | A methodological quality assessment of systematic reviews and meta-analyses of antidepressants effect on low back pain using updated AMSTAR | Panahi et al. 2020 | 31973739 | <https://pubmed.ncbi.nlm.nih.gov/31973739/> | Evidence-Based Recommendations on the Pharmacological Management of Osteoarthritis and Chronic Low Back Pain: An Asian Consensus |  |
| 2 | 2 | A Systematic Review and Best Evidence Synthesis of the Effectiveness of Therapeutic Facet Joint Interventions in Managing Chronic Spinal Pain | Manchikanti et al. 2015 | 26218948 | <https://pubmed.ncbi.nlm.nih.gov/26218948/> | Comprehensive Evidence-Based Guidelines for Facet Joint Interventions in the Management of Chronic Spinal Pain: American Society of Interventional Pain Physicians (ASIPP) Guidelines  Responsible, Safe, and Effective Use of Biologics in the Management of Low Back Pain: American Society of Interventional Pain Physicians (ASIPP) Guidelines |  |
| 3 | 3 | A systematic review and meta-analysis of efficacy, cost-effectiveness, and safety of selected complementary and alternative medicine for neck and low-back pain | Furlan et al. 2011 | 22203884 | <https://pubmed.ncbi.nlm.nih.gov/22203884/> | Comprehensive Evidence-Based Guidelines for Facet Joint Interventions in the Management of Chronic Spinal Pain: American Society of Interventional Pain Physicians (ASIPP) Guidelines  Spinal Manipulative Therapy and Other Conservative Treatments for Low Back Pain: A Guideline From the Canadian Chiropractic Guideline Initiative |  |
| 4 | 4 | A systematic review and meta-analysis of yoga for low back pain | Cramer et al. 2013 | 23246998 | <https://pubmed.ncbi.nlm.nih.gov/23246998/> | Noninvasive Treatments for Acute, Subacute, and Chronic Low Back Pain: A Clinical Practice Guideline From the American College of Physicians |  |
| 5 | 5 | A Systematic Review of Mesenchymal Stem Cells in Spinal Cord Injury, Intervertebral Disc Repair and Spinal Fusion | Khan et al. 2018 | 28891440 | <https://pubmed.ncbi.nlm.nih.gov/28891440/> | Responsible, Safe, and Effective Use of Biologics in the Management of Low Back Pain: American Society of Interventional Pain Physicians (ASIPP) Guidelines |  |
| 6 | 6 | A systematic review on the effectiveness of physical and rehabilitation interventions for chronic non- specific low back pain | Middelkoop et al. 2010 | 20640863 | <https://pubmed.ncbi.nlm.nih.gov/20640863/> | Noninvasive Treatments for Acute, Subacute, and Chronic Low Back Pain: A Clinical Practice Guideline From the American College of Physicians |  |
| 7 | 7 | Acupuncture for acute low back pain: a systematic review | Lee et al. 2013 | 23269281 | <https://pubmed.ncbi.nlm.nih.gov/23269281/> | Noninvasive Treatments for Acute, Subacute, and Chronic Low Back Pain: A Clinical Practice Guideline From the American College of Physicians |  |
| 8 | 8 | Antidepressants for non-specific low back pain | Urquhart et al. 2008 | 18253994 | <https://pubmed.ncbi.nlm.nih.gov/18253994/> | Noninvasive Treatments for Acute, Subacute, and Chronic Low Back Pain: A Clinical Practice Guideline From the American College of Physicians |  |
| 9 | 9 | Antineuropathic and antinociceptive drugs combination in patients with chronic low back pain: a systematic review | Romano et al. 2012 | 22619711 | <https://pubmed.ncbi.nlm.nih.gov/22619711/> | Evidence-Based Recommendations on the Pharmacological Management of Osteoarthritis and Chronic Low Back Pain: An Asian Consensus |  |
| 10 | 10 | Are non-steroidal anti-inflammatory drugs effective for the management of neck pain and associated disorders, whiplash-associated disorders, or non- specific low back pain? A systematic review of systematic reviews by the Ontario Protocol for Traffic Injury Management (OPTIMa) Collaboration | Wong et al. 2015 | 25827308 | <https://pubmed.ncbi.nlm.nih.gov/25827308/> | Evidence-Based Recommendations on the Pharmacological Management of Osteoarthritis and Chronic Low Back Pain: An Asian Consensus |  |
| 11 | 11 | Association of Spinal Manipulative Therapy With Clinical Benefit and Harm for Acute Low Back Pain: Systematic Review and Meta-analysis | Paige et al. 2017 | 28399251 | <https://pubmed.ncbi.nlm.nih.gov/28399251/> | Spinal Manipulative Therapy and Other Conservative Treatments for Low Back Pain: A Guideline From the Canadian Chiropractic Guideline Initiative |  |
| 12 | 12 | Behavioural treatment for chronic low-back pain | Henschke et al. 2010 | 20614428 | <https://pubmed.ncbi.nlm.nih.gov/20614428/> | Noninvasive Treatments for Acute, Subacute, and Chronic Low Back Pain: A Clinical Practice Guideline From the American College of Physicians |  |
| 13 | 13 | Cell-Based Therapies for Lumbar Discogenic Low Back Pain: Systematic Review and Single-Arm Meta- analysis | Wu et al. 2018 | 26953666 | <https://pubmed.ncbi.nlm.nih.gov/26953666/> | Responsible, Safe, and Effective Use of Biologics in the Management of Low Back Pain: American Society of Interventional Pain Physicians (ASIPP) Guidelines |  |
| 14 | 14 | Clinical effectiveness of manual therapy for the management of musculoskeletal and non- musculoskeletal conditions: systematic review and update of UK evidence report | Clar et al. 2014 | 24679336 | <https://pubmed.ncbi.nlm.nih.gov/24679336/> | Best Practices for Chiropractic Management of Patients with Chronic Musculoskeletal Pain: A Clinical Practice Guideline |  |

| 15 | 15 | Clinical practice guidelines for the noninvasive management of low back pain: A systematic review by the Ontario Protocol for Traffic Injury Management (OPTIMa) Collaboration | Wong et al. 2016 | 27712027 | <https://pubmed.ncbi.nlm.nih.gov/27712027/> | Spinal Manipulative Therapy and Other Conservative Treatments for Low Back Pain: A Guideline From the Canadian Chiropractic Guideline Initiative |  |
| --- | --- | --- | --- | --- | --- | --- | --- |
| 16 | 16 | Comparison of Clinical Efficacy Between Transforaminal and Interlaminar Epidural Injections in Lumbosacral Disc Herniation: A Systematic Review and Meta-Analysis | Lee et al. 2018 | 30282389 | <https://pubmed.ncbi.nlm.nih.gov/30282389/> | Comprehensive Evidence-Based Guidelines for Facet Joint Interventions in the Management  of Chronic Spinal Pain: American Society of Interventional Pain Physicians (ASIPP) Guidelines  Nonsurgical treatments for patients with radicular pain from lumbosacral disc herniation  Responsible, Safe, and Effective Use of Biologics in the Management of Low Back Pain: American Society of Interventional Pain Physicians (ASIPP) Guidelines |  |
| 17 | 17 | Comparison of Clinical Efficacy of Epidural Injection With or Without Steroid in Lumbosacral Disc Herniation: A Systematic Review and Meta-analysis | Lee et al. 2018 | 30282390 | <https://pubmed.ncbi.nlm.nih.gov/30282390/> | Comprehensive Evidence-Based Guidelines for Facet Joint Interventions in the Management  of Chronic Spinal Pain: American Society of Interventional Pain Physicians (ASIPP) Guidelines  Responsible, Safe, and Effective Use of Biologics in the Management of Low Back Pain: American Society of Interventional Pain Physicians (ASIPP) Guidelines |  |
| 18 | 18 | Comparison of clinical efficacy of transforaminal and caudal epidural steroid injection in lumbar and lumbosacral disc herniation: A systematic review and meta-analysis | Lee et al. 2018 | 30030083 | <https://pubmed.ncbi.nlm.nih.gov/30030083/> | Nonsurgical treatments for patients with radicular pain from lumbosacral disc herniation |  |
| 19 | 19 | Comparison of the efficacy of saline, local anesthetics, and steroids in epidural and facet joint injections for the management of spinal pain: A systematic review of randomized controlled trials | Manchikanti et al. 2015 | 26005584 | <https://pubmed.ncbi.nlm.nih.gov/26005584/> | Comprehensive Evidence-Based Guidelines for Facet Joint Interventions in the Management  of Chronic Spinal Pain: American Society of Interventional Pain Physicians (ASIPP) Guidelines  Responsible, Safe, and Effective Use of Biologics in the Management of Low Back Pain: American Society of Interventional Pain Physicians (ASIPP) Guidelines |  |
| 20 | 20 | Conservative management of lumbar disc herniation with associated radiculopathy: a systematic review | Hahne et al. 2010 | 20421859 | <https://pubmed.ncbi.nlm.nih.gov/20421859/> | Nonsurgical treatments for patients with radicular pain from lumbosacral disc herniation  Responsible, Safe, and Effective Use of Biologics in the Management of Low Back Pain: American Society of Interventional Pain Physicians (ASIPP) Guidelines |  |
| 21 | 21 | Do Epidural Injections Provide Short- and Long-term Relief for Lumbar Disc Herniation? A Systematic Review | Manchikanti et al. 2015 | 24515404 | <https://pubmed.ncbi.nlm.nih.gov/24515404/> | Nonsurgical treatments for patients with radicular pain from lumbosacral disc herniation  Responsible, Safe, and Effective Use of Biologics in the Management of Low Back Pain: American Society of Interventional Pain Physicians (ASIPP) Guidelines |  |
| 22 | 22 | Do Regenerative Medicine Therapies Provide Long- Term Relief in Chronic Low Back Pain: A Systematic Review and Metaanalysis | Sanapati et al. 2018 | 30508983 | <https://pubmed.ncbi.nlm.nih.gov/30508983/> | Responsible, Safe, and Effective Use of Biologics in the Management of Low Back Pain: American Society of Interventional Pain Physicians (ASIPP) Guidelines |  |
| 23 | 23 | Does Epidural Bupivacaine with or Without Steroids Provide Long-Term Relief? A Systematic Review and Meta-analysis | Manchikanti et al. 2020 | 32335757 | <https://pubmed.ncbi.nlm.nih.gov/32335757/> | Comprehensive Evidence-Based Guidelines for Facet Joint Interventions in the Management of Chronic Spinal Pain: American Society of Interventional Pain Physicians (ASIPP) Guidelines |  |
| 24 | 24 | Drug therapy for the treatment of chronic nonspecific low back pain: systematic review and meta-analysis | Chung et al. 2013 | 24284847 | <https://pubmed.ncbi.nlm.nih.gov/24284847/> | Evidence-Based Recommendations on the Pharmacological Management of Osteoarthritis and Chronic Low Back Pain: An Asian Consensus |  |
| 25 | 25 | Effectiveness and Economic Evaluation of Chiropractic Care for the Treatment of Low Back Pain: A Systematic Review of Pragmatic Studies | Blanchette et al. 2016 | 27487116 | <https://pubmed.ncbi.nlm.nih.gov/27487116/> | Spinal Manipulative Therapy and Other Conservative Treatments for Low Back Pain: A Guideline From the Canadian Chiropractic Guideline Initiative |  |

| 26 | 26 | Effectiveness of acupuncture for nonspecific chronic low back pain: a systematic review and meta-analysis | Lam et al. 2013 | 24026151 | <https://pubmed.ncbi.nlm.nih.gov/24026151/> | Noninvasive Treatments for Acute, Subacute, and Chronic Low Back Pain: A Clinical Practice Guideline From the American College of Physicians |  |
| --- | --- | --- | --- | --- | --- | --- | --- |
| 27 | 27 | Effectiveness of Percutaneous Adhesiolysis in Managing Chronic Central Lumbar Spinal Stenosis: A Systematic Review and Meta-Analysis | Manchikanti et al. 2019 | 31775400 | <https://pubmed.ncbi.nlm.nih.gov/31775400/> | Comprehensive Evidence-Based Guidelines for Facet Joint Interventions in the Management of Chronic Spinal Pain: American Society of Interventional Pain Physicians (ASIPP) Guidelines |  |
| 28 | 28 | Effectiveness of Percutaneous Adhesiolysis in Post Lumbar Surgery Syndrome: A Systematic Analysis of Findings of Systematic Reviews | Manchikanti et al. 2019 | 31337160 | <https://pubmed.ncbi.nlm.nih.gov/31337160/> | Comprehensive Evidence-Based Guidelines for Facet Joint Interventions in the Management of Chronic Spinal Pain: American Society of Interventional Pain Physicians (ASIPP) Guidelines |  |
| 29 | 29 | Effectiveness of Spinal Cord Stimulation in Chronic Spinal Pain: A Systematic Review | Grider et al. 2016 | 26752493 | <https://pubmed.ncbi.nlm.nih.gov/26752493/> | Responsible, Safe, and Effective Use of Biologics in the Management of Low Back Pain: American Society of Interventional Pain Physicians (ASIPP) Guidelines |  |
| 30 | 30 | Efficacy and safety of paracetamol for spinal pain and osteoarthritis: systematic review and meta-analysis of randomised placebo controlled trials | Machado et al. 2015 | 25828856 | <https://pubmed.ncbi.nlm.nih.gov/25828856/> | Evidence-Based Recommendations on the Pharmacological Management of Osteoarthritis and Chronic Low Back Pain: An Asian Consensus |  |
| 31 | 31 | Efficacy and tolerance of systemic steroids in sciatica: a systematic review and meta-analysis | Roncoroni et al. 2011 | 21525139 | <https://pubmed.ncbi.nlm.nih.gov/21525139/> | Nonsurgical treatments for patients with radicular pain from lumbosacral disc herniation |  |
| 32 | 32 | Efficacy of epidural injections in the treatment of lumbar central spinal stenosis: a systematic review | Manchikanti et al. 2015 | 25789241 | <https://pubmed.ncbi.nlm.nih.gov/25789241/> | Responsible, Safe, and Effective Use of Biologics in the Management of Low Back Pain: American Society of Interventional Pain Physicians (ASIPP) Guidelines |  |
| 33 | 33 | Efficacy, Tolerability, and Dose-Dependent Effects of Opioid Analgesics for Low Back Pain: A Systematic Review and Meta-analysis | Shaheed et al. 2016 | 27213267 | <https://pubmed.ncbi.nlm.nih.gov/27213267/> | Evidence-Based Recommendations on the Pharmacological Management of Osteoarthritis and Chronic Low Back Pain: An Asian Consensus |  |
| 34 | 34 | Epidural Corticosteroid Injections for Radiculopathy and Spinal Stenosis: A Systematic Review and Meta- analysis | Chou et al. 2015 | 26302454 | <https://pubmed.ncbi.nlm.nih.gov/26302454/> | Comprehensive Evidence-Based Guidelines for Facet Joint Interventions in the Management  of Chronic Spinal Pain: American Society of Interventional Pain Physicians (ASIPP) Guidelines  Responsible, Safe, and Effective Use of Biologics in the Management of Low Back Pain: American Society of Interventional Pain Physicians (ASIPP) Guidelines |  |
| 35 | 35 | Epidural injection with or without steroid in managing chronic low back and lower extremity pain: ameta- analysis of ten randomized controlled trials | Zhai et al. 2015 | 26309483 | <https://pubmed.ncbi.nlm.nih.gov/26309483/> | Nonsurgical treatments for patients with radicular pain from lumbosacral disc herniation  Responsible, Safe, and Effective Use of Biologics in the Management of Low Back Pain: American Society of Interventional Pain Physicians (ASIPP) Guidelines |  |
| 36 | 36 | Epidural Injection With or Without Steroid in Managing Chronic Low-Back and Lower Extremity Pain: A Meta-Analysis of 10 Randomized Controlled Trials | Zhai et al. 2017 | 26035031 | <https://pubmed.ncbi.nlm.nih.gov/26035031/> | Responsible, Safe, and Effective Use of Biologics in the Management of Low Back Pain: American Society of Interventional Pain Physicians (ASIPP) Guidelines |  |
| 37 | 37 | Epidural Injections for Lumbar Radiculopathy and Spinal Stenosis: A Comparative Systematic Review and Meta-Analysis | Manchikanti et al. 2016 | 27008296 | <https://pubmed.ncbi.nlm.nih.gov/27008296/> | Comprehensive Evidence-Based Guidelines for Facet Joint Interventions in the Management  of Chronic Spinal Pain: American Society of Interventional Pain Physicians (ASIPP) Guidelines  Responsible, Safe, and Effective Use of Biologics in the Management of Low Back Pain: American Society of Interventional Pain Physicians (ASIPP) Guidelines |  |
| 38 | 38 | Epidural injections in prevention of surgery for spinal pain: systematic review and meta-analysis of randomized controlled trials | Bicket et al. 2014 | 25463400 | <https://pubmed.ncbi.nlm.nih.gov/25463400/> | Nonsurgical treatments for patients with radicular pain from lumbosacral disc herniation |  |
| 39 | 39 | Epidural injections with or without steroids in managing chronic low back pain secondary to lumbar spinal stenosis: a meta-analysis of 13 randomized controlled trials | Meng et al. 2015 | 26316704 | <https://pubmed.ncbi.nlm.nih.gov/26316704/> | Responsible, Safe, and Effective Use of Biologics in the Management of Low Back Pain: American Society of Interventional Pain Physicians (ASIPP) Guidelines |  |
| 40 | 40 | Epidural steroid injections for radicular lumbosacral pain: a systematic review | Shamliyan et al. 2014 | 24787344 | <https://pubmed.ncbi.nlm.nih.gov/24787344/> | Nonsurgical treatments for patients with radicular pain from lumbosacral disc herniation |  |

| 41 | 41 | Exercise for the prevention of low back and pelvic girdle pain in pregnancy: A meta-analysis of randomized controlled trials | Shiri et al. 2017 | 28869318 | <https://pubmed.ncbi.nlm.nih.gov/28869318/> | Spinal Manipulative Therapy and Other Conservative Treatments for Low Back Pain: A Guideline From the Canadian Chiropractic Guideline Initiative |  |
| --- | --- | --- | --- | --- | --- | --- | --- |
| 42 | 42 | Fusion or Not for Degenerative Lumbar Spinal Stenosis: A Meta-Analysis and Systematic Review | Shen et al. 2018 | 29357326 | <https://pubmed.ncbi.nlm.nih.gov/29357326/> | Comprehensive Evidence-Based Guidelines for Facet Joint Interventions in the Management  of Chronic Spinal Pain: American Society of Interventional Pain Physicians (ASIPP) Guidelines  Responsible, Safe, and Effective Use of Biologics in the Management of Low Back Pain: American Society of Interventional Pain Physicians (ASIPP) Guidelines |  |
| 43 | 43 | Literature Review and Meta-Analysis of Transcutaneous Electrical Nerve Stimulation in Treating Chronic Back Pain | Wu et al. 2018 | 29394211 | <https://pubmed.ncbi.nlm.nih.gov/29394211/> | Best Practices for Chiropractic Management of Patients with Chronic Musculoskeletal Pain: A Clinical Practice Guideline |  |
| 44 | 44 | Massage for low-back pain | Furlan et al. 2008 | 18843627 | <https://pubmed.ncbi.nlm.nih.gov/18843627/> | Noninvasive Treatments for Acute, Subacute, and Chronic Low Back Pain: A Clinical Practice Guideline From the American College of Physicians |  |
| 45 | 45 | Medial branch neurotomy in management of chronic spinal pain: systematic review of the evidence | Manchikanti et al. 2002 | 16886020 | <https://pubmed.ncbi.nlm.nih.gov/16886020/> | Comprehensive Evidence-Based Guidelines for Facet Joint Interventions in the Management  of Chronic Spinal Pain: American Society of Interventional Pain Physicians (ASIPP) Guidelines |  |
| 46 | 46 | Meta-analysis: acupuncture for low back pain | Manheimer et al. 2005 | 15838072 | <https://pubmed.ncbi.nlm.nih.gov/15838072/> | Noninvasive Treatments for Acute, Subacute, and Chronic Low Back Pain: A Clinical Practice Guideline From the American College of Physicians |  |
| 47 | 47 | Motor control exercises reduces pain and disability in chronic and recurrent low back pain: a meta-analysis | Bystrom et al. 2013 | 23492976 | <https://pubmed.ncbi.nlm.nih.gov/23492976/> | Noninvasive Treatments for Acute, Subacute, and Chronic Low Back Pain: A Clinical Practice Guideline From the American College of Physicians |  |
| 48 | 48 | Multidisciplinary biopsychosocial rehabilitation for chronic low back pain | Kamper et al. 2014 | 25180773 | <https://pubmed.ncbi.nlm.nih.gov/25180773/> | Noninvasive Treatments for Acute, Subacute, and Chronic Low Back Pain: A Clinical Practice Guideline From the American College of Physicians |  |
| 49 | 49 | Multidisciplinary biopsychosocial rehabilitation for chronic low back pain: Cochrane systematic review and meta-analysis | Kamper et al. 2015 | 25694111 | <https://pubmed.ncbi.nlm.nih.gov/25694111/> | Best Practices for Chiropractic Management of Patients with Chronic Musculoskeletal Pain: A Clinical Practice Guideline |  |
| 50 | 50 | Muscle relaxants for non-specific low back pain | Tulder et al. 2003 | 12804507 | <https://pubmed.ncbi.nlm.nih.gov/12804507/> | Noninvasive Treatments for Acute, Subacute, and Chronic Low Back Pain: A Clinical Practice Guideline From the American College of Physicians |  |
| 51 | 51 | Noninvasive Nonpharmacological Treatment for Chronic Pain: A Systematic Review [Internet] | Skelly et al. 2018 | 30179389 | <https://pubmed.ncbi.nlm.nih.gov/30179389/> | Comprehensive Evidence-Based Guidelines for Facet Joint Interventions in the Management of Chronic Spinal Pain: American Society of Interventional Pain Physicians (ASIPP) Guidelines |  |
| 52 | 52 | Noninvasive Nonpharmacological Treatment for Chronic Pain: A Systematic Review Update [Internet] | Skelly et al. 2020 | 32338846 | <https://pubmed.ncbi.nlm.nih.gov/32338846/> | Best Practices for Chiropractic Management of Patients with Chronic Musculoskeletal Pain: A Clinical Practice Guideline  Comprehensive Evidence-Based Guidelines for Facet Joint Interventions in the Management of Chronic Spinal Pain: American Society of Interventional Pain Physicians (ASIPP) Guidelines |  |
| 53 | 53 | Nonpharmacologic therapies for acute and chronic low back pain: a review of the evidence for an American Pain Society/American College of Physicians clinical practice guideline | Chou et al. 2007 | 17909210 | <https://pubmed.ncbi.nlm.nih.gov/17909210/> | Nonsurgical treatments for patients with radicular pain from lumbosacral disc herniation  Spinal Manipulative Therapy and Other Conservative Treatments for Low Back Pain: A Guideline From the Canadian Chiropractic Guideline Initiative |  |

| 54 | 54 | Nonpharmacologic Therapies for Low Back Pain: A Systematic Review for an American College of Physicians Clinical Practice Guideline | Chou et al. 2017 | 28192793 | <https://pubmed.ncbi.nlm.nih.gov/28192793/> | JAMA Clinical Guidelines Synopsis Treatment of Low Back Pain  Noninvasive Treatments for Acute, Subacute, and Chronic Low Back Pain: A Clinical Practice Guideline From the American College of Physicians  Spinal Manipulative Therapy and Other Conservative Treatments for Low Back Pain: A Guideline From the Canadian Chiropractic Guideline Initiative |  |
| --- | --- | --- | --- | --- | --- | --- | --- |
| 55 | 55 | Non-steroidal anti-inflammatory drugs for chronic low back pain | Enthoven et al. 2016 | 26863524 | <https://pubmed.ncbi.nlm.nih.gov/26863524/> | Evidence-Based Recommendations on the Pharmacological Management of Osteoarthritis and Chronic Low Back Pain: An Asian Consensus |  |
| 56 | 56 | Non-steroidal anti-inflammatory drugs for low back pain | Roelofs et al. 2008 | 18253976 | <https://pubmed.ncbi.nlm.nih.gov/18253976/> | Noninvasive Treatments for Acute, Subacute, and Chronic Low Back Pain: A Clinical Practice Guideline From the American College of Physicians |  |
| 57 | 57 | Non-steroidal anti-inflammatory drugs for sciatica | Rasmussen-Barr et al. 2016 | 27743405 | <https://pubmed.ncbi.nlm.nih.gov/27743405/> | Nonsurgical treatments for patients with radicular pain from lumbosacral disc herniation |  |
| 58 | 58 | Opioids compared to placebo or other treatments for chronic low-back pain | Chaparro et al. 2013 | 23983011 | <https://pubmed.ncbi.nlm.nih.gov/23983011/> | Evidence-Based Recommendations on the Pharmacological Management of Osteoarthritis and Chronic Low Back Pain: An Asian Consensus  Noninvasive Treatments for Acute, Subacute, and Chronic Low Back Pain: A Clinical Practice Guideline From the American College of Physicians |  |
| 59 | 59 | Percutaneous and Endoscopic Adhesiolysis in Managing Low Back and Lower Extremity Pain: A Systematic Review and Meta-analysis | Helm et al. 2016 | 26815254 | <https://pubmed.ncbi.nlm.nih.gov/26815254/> | Responsible, Safe, and Effective Use of Biologics in the Management of Low Back Pain: American Society of Interventional Pain Physicians (ASIPP) Guidelines |  |
| 60 | 60 | Physical activity and exercise for chronic pain in adults: an overview of Cochrane Reviews | Geneen et al. 2017 | 28087891 | <https://pubmed.ncbi.nlm.nih.gov/28087891/> | Spinal Manipulative Therapy and Other Conservative Treatments for Low Back Pain: A Guideline From the Canadian Chiropractic Guideline Initiative |  |
| 61 | 61 | Prevention of Low Back Pain: A Systematic Review and Meta-analysis | Steffens et al. 2016 | 26752509 | <https://pubmed.ncbi.nlm.nih.gov/26752509/> | Nonsurgical treatments for patients with radicular pain from lumbosacral disc herniation  Spinal Manipulative Therapy and Other Conservative Treatments for Low Back Pain: A Guideline From the Canadian Chiropractic Guideline Initiative |  |
| 62 | 62 | Radiofrequency ablation for chronic low back pain: a systematic review of randomized controlled trials | Leggett et al. 2014 | 25068973 | <https://pubmed.ncbi.nlm.nih.gov/25068973/> | Comprehensive Evidence-Based Guidelines for Facet Joint Interventions in the Management of Chronic Spinal Pain: American Society of Interventional Pain Physicians (ASIPP) Guidelines |  |
| 63 | 63 | Radiofrequency denervation for chronic low back pain | Mass et al. 2015 | 26495910 | <https://pubmed.ncbi.nlm.nih.gov/26495910/> | Comprehensive Evidence-Based Guidelines for Facet Joint Interventions in the Management of Chronic Spinal Pain: American Society of Interventional Pain Physicians (ASIPP) Guidelines |  |
| 64 | 64 | Radiofrequency denervation for facet joint low back pain: a systematic review | Poetscher et al. 2014 | 24732848 | <https://pubmed.ncbi.nlm.nih.gov/24732848/> | Comprehensive Evidence-Based Guidelines for Facet Joint Interventions in the Management of Chronic Spinal Pain: American Society of Interventional Pain Physicians (ASIPP) Guidelines |  |
| 65 | 65 | Role of Epidural Injections to Prevent Surgical Intervention in Patients with Chronic Sciatica: A Systematic Review and Meta-Analysis | Bhatti et al. 2016 | 27625909 | <https://pubmed.ncbi.nlm.nih.gov/27625909/> | Nonsurgical treatments for patients with radicular pain from lumbosacral disc herniation |  |
| 66 | 66 | Sacroiliac Joint Fusion Methodology - Minimally Invasive Compared to Screw-Type Surgeries: A Systematic Review and Meta-Analysis | Tran et al. 2019 | 30700066 | <https://pubmed.ncbi.nlm.nih.gov/30700066/> | Comprehensive Evidence-Based Guidelines for Facet Joint Interventions in the Management of Chronic Spinal Pain: American Society of Interventional Pain Physicians (ASIPP) Guidelines |  |
| 67 | 67 | Serious Adverse Events and Spinal Manipulative Therapy of the Low Back Region: A Systematic Review of Cases | Hebert et al. 2013 | 23787298 | <https://pubmed.ncbi.nlm.nih.gov/23787298/> | Spinal Manipulative Therapy and Other Conservative Treatments for Low Back Pain: A Guideline From the Canadian Chiropractic Guideline Initiative |  |
| 68 | 68 | Sling exercise for chronic low back pain: a systematic review and meta-analysis | Yue et al. 2014 | 24919119 | <https://pubmed.ncbi.nlm.nih.gov/24919119/> | Nonsurgical treatments for patients with radicular pain from lumbosacral disc herniation |  |

| 69 | 69 | Spinal manipulative therapy for acute low-back pain | Rubinstein et al. 2012 | 22972127 | <https://pubmed.ncbi.nlm.nih.gov/22972127/> | Noninvasive Treatments for Acute, Subacute, and Chronic Low Back Pain: A Clinical Practice Guideline From the American College of Physicians |  |
| --- | --- | --- | --- | --- | --- | --- | --- |
| 70 | 70 | Spinal manipulative therapy for chronic low-back pain | Rubinstein et al. 2011 | 21328304 | <https://pubmed.ncbi.nlm.nih.gov/21328304/> | Noninvasive Treatments for Acute, Subacute, and Chronic Low Back Pain: A Clinical Practice Guideline From the American College of Physicians |  |
| 71 | 71 | Spinal manipulative therapy for chronic low-back pain: an update of a Cochrane review | Rubinstein et al. 2011 | 21593658 | <https://pubmed.ncbi.nlm.nih.gov/21593658/> | Spinal Manipulative Therapy and Other Conservative Treatments for Low Back Pain: A Guideline From the Canadian Chiropractic Guideline Initiative |  |
| 72 | 72 | Steroid for epidural injection in spinal stenosis: a systematic review and meta-analysis | Liu et al. 2015 | 25678775 | <https://pubmed.ncbi.nlm.nih.gov/25678775/> | Responsible, Safe, and Effective Use of Biologics in the Management of Low Back Pain: American Society of Interventional Pain Physicians (ASIPP) Guidelines |  |
| 73 | 73 | Superficial heat or cold for low back pain | French et al. 2006 | 16437495 | <https://pubmed.ncbi.nlm.nih.gov/16437495/> | Noninvasive Treatments for Acute, Subacute, and Chronic Low Back Pain: A Clinical Practice Guideline From the American College of Physicians |  |
| 74 | 74 | Systematic review of effectiveness and complications of adhesiolysis in the management of chronic spinal pain: an update | Trescot et al. 2007 | 17256027 | <https://pubmed.ncbi.nlm.nih.gov/17256027/> | Nonsurgical treatments for patients with radicular pain from lumbosacral disc herniation |  |
| 75 | 75 | Systematic Review of the Diagnostic Accuracy and Therapeutic Effectiveness of Sacroiliac Joint Interventions | Simopoulos et al. 2015 | 26431129 | <https://pubmed.ncbi.nlm.nih.gov/26431129/> | Comprehensive Evidence-Based Guidelines for Facet Joint Interventions in the Management of Chronic Spinal Pain: American Society of Interventional Pain Physicians (ASIPP) Guidelines  Responsible, Safe, and Effective Use of Biologics in the Management of Low Back Pain: American Society of Interventional Pain Physicians (ASIPP) Guidelines |  |
| 76 | 76 | Systematic Review of the Effectiveness of Lumbar Medial Branch Thermal Radiofrequency Neurotomy, Stratified for Diagnostic Methods and Procedural Technique | Schneider et al. 2020 | 32040149 | <https://pubmed.ncbi.nlm.nih.gov/32040149/> | Comprehensive Evidence-Based Guidelines for Facet Joint Interventions in the Management of Chronic Spinal Pain: American Society of Interventional Pain Physicians (ASIPP) Guidelines |  |
| 77 | 77 | Systemic Pharmacologic Therapies for Low Back Pain: A Systematic Review for an American College of Physicians Clinical Practice Guideline | Chou et al. 2017 | 28192790 | <https://pubmed.ncbi.nlm.nih.gov/28192790/> | JAMA Clinical Guidelines Synopsis: Treatment of Low Back Pain  Noninvasive Treatments for Acute, Subacute, and Chronic Low Back Pain: A Clinical Practice Guideline From the American College of Physicians |  |
| 78 | 78 | Tapentadol for chronic musculoskeletal pain in adults | Santos et al 2015 | 26017279 | <https://pubmed.ncbi.nlm.nih.gov/26017279/> | Evidence-Based Recommendations on the Pharmacological Management of Osteoarthritis and Chronic Low Back Pain: An Asian Consensus |  |
| 79 | 79 | The effect of antidepressant treatment on chronic back pain: a meta-analysis | Salerno et al. 2002 | 11784215 | <https://pubmed.ncbi.nlm.nih.gov/11784215/> | Noninvasive Treatments for Acute, Subacute, and Chronic Low Back Pain: A Clinical Practice Guideline From the American College of Physicians |  |
| 80 | 80 | The effectiveness of Pilates exercise in people with chronic low back pain: a systematic review | Wells et al. 2014 | 24984069 | <https://pubmed.ncbi.nlm.nih.gov/24984069/> | Noninvasive Treatments for Acute, Subacute, and Chronic Low Back Pain: A Clinical Practice Guideline From the American College of Physicians |  |
| 81 | 81 | The effectiveness of walking versus exercise on pain and function in chronic low back pain: a systematic review and meta-analysis of randomized trials | Vanti et al. 2017 | 29207885 | <https://pubmed.ncbi.nlm.nih.gov/29207885/> | Nonsurgical treatments for patients with radicular pain from lumbosacral disc herniation |  |
| 82 | 82 | The efficacy of conventional radiofrequency denervation in patients with chronic low back pain originating from the facet joints: a meta-analysis of randomized controlled trials | Lee et al. 2017 | 28576500 | <https://pubmed.ncbi.nlm.nih.gov/28576500/> | Comprehensive Evidence-Based Guidelines for Facet Joint Interventions in the Management of Chronic Spinal Pain: American Society of Interventional Pain Physicians (ASIPP) Guidelines |  |
| 83 | 83 | Therapeutic ultrasound for chronic low back pain | Ebadi et al. 2020 | 32623724 | <https://pubmed.ncbi.nlm.nih.gov/32623724/> | Noninvasive Treatments for Acute, Subacute, and Chronic Low Back Pain: A Clinical Practice Guideline From the American College of Physicians |  |
| 84 | 84 | Therapeutic ultrasound for chronic low-back pain | Ebadi et al. 2014 | 24627326 | <https://pubmed.ncbi.nlm.nih.gov/24627326/> | Noninvasive Treatments for Acute, Subacute, and Chronic Low Back Pain: A Clinical Practice Guideline From the American College of Physicians |  |

| 85 | 85 | Topical NSAIDs for chronic musculoskeletal pain in adults | Derry et al. 2016 | 27103611 | <https://pubmed.ncbi.nlm.nih.gov/27103611/> | Evidence-Based Recommendations on the Pharmacological Management of Osteoarthritis and Chronic Low Back Pain: An Asian Consensus |  |
| --- | --- | --- | --- | --- | --- | --- | --- |
| 86 | 86 | Traction for low-back pain with or without sciatica | Weger et al. 2013 | 23959683 | <https://pubmed.ncbi.nlm.nih.gov/23959683/> | Noninvasive Treatments for Acute, Subacute, and Chronic Low Back Pain: A Clinical Practice Guideline From the American College of Physicians |  |
| 87 | 87 | Transcutaneous electrical nerve stimulation and interferential current demonstrate similar effects in relieving acute and chronic pain: a systematic review with meta-analysis | Almeida et al. 2018 | 29426587 | <https://pubmed.ncbi.nlm.nih.gov/29426587/> | Best Practices for Chiropractic Management of Patients with Chronic Musculoskeletal Pain: A Clinical Practice Guideline |  |
| 88 | 88 | Transforaminal versus interlaminar approaches to epidural steroid injections: a systematic review of comparative studies for lumbosacral radicular pain | Chang-Chien et al. 2014 | 25054401 | <https://pubmed.ncbi.nlm.nih.gov/25054387/> | Nonsurgical treatments for patients with radicular pain from lumbosacral disc herniation |  |
| 89 | 89 | What is the Role of Epidural Injections in the Treatment of Lumbar Discogenic Pain: A Systematic Review of Comparative Analysis with Fusion | Manchikanti et al. 2015 | 25852828 | <https://pubmed.ncbi.nlm.nih.gov/25852828/> | Responsible, Safe, and Effective Use of Biologics in the Management of Low Back Pain: American Society of Interventional Pain Physicians (ASIPP) Guidelines |  |
| 90 | 90 | Amitriptyline for musculoskeletal complaints: a systematic review | Driest et al. 2017 | 28334783 | <https://pubmed.ncbi.nlm.nih.gov/28334783/> | Evidence-Based Recommendations on the Pharmacological Management of Osteoarthritis and Chronic Low Back Pain: An Asian Consensus |  |
| 91 | 1 | Exercise for the Prevention of Low Back Pain: Systematic Review and Meta-Analysis of Controlled Trials | Shiri et al. 2018 | 29053873 | <https://pubmed.ncbi.nlm.nih.gov/29053873/> | Out of Scope | 1 |
| 92 | 2 | Medications for acute and chronic low back pain: a review of the evidence for an American Pain Society/American College of Physicians clinical practice guideline | Chou et al. 2007 | 17909211 | <https://pubmed.ncbi.nlm.nih.gov/17909211/> | Out of Scope | 2 |
| 93 | 3 | Spinal Manipulation Vs Sham Manipulation for Nonspecific Low Back Pain: A Systematic Review and Meta-analysis | Ruddock et al. 2016 | 27660593 | <https://pubmed.ncbi.nlm.nih.gov/27660593/> | Out of Scope | 3 |
| 94 | 4 | Pain Catastrophizing and Function In Individuals With Chronic Musculoskeletal Pain: A Systematic Review and Meta-Analysis | Martinez-Calderon et al.  2019 | 30664551 | <https://pubmed.ncbi.nlm.nih.gov/30664551/> | Out of Scope | 4 |
| 95 | 5 | Poor overall quality of clinical practice guidelines for musculoskeletal pain: a systematic review | Lin et al. 2017 | 29175827 | <https://pubmed.ncbi.nlm.nih.gov/29175827/> | Out of Scope | 5 |
| 96 | 6 | Patient and public attitudes to and awareness of clinical practice guidelines: a systematic review with thematic and narrative syntheses | Loudon et al. 2014 | 25064372 | <https://pubmed.ncbi.nlm.nih.gov/25064372/> | Out of Scope | 6 |
| 97 | 7 | Efficacy of intervertebral disc regeneration with stem cells - a systematic review and meta-analysis of animal controlled trials | Wang et al. 2015 | 25796605 | <https://pubmed.ncbi.nlm.nih.gov/25796605/> | Out of Scope | 7 |
| 98 | 8 | AMSTAR is a reliable and valid measurement tool to assess the methodological quality of systematic reviews | Shea et al. 2009 | 19230606 | <https://pubmed.ncbi.nlm.nih.gov/19230606/> | Out of Scope | 8 |
| 99 | 9 | Are Pain Beliefs, Cognitions, and Behaviors Influenced by Race, Ethnicity, and Culture in Patients with Chronic Musculoskeletal Pain: A Systematic Review | Orhan et al. 2018 | 30508984 | <https://pubmed.ncbi.nlm.nih.gov/30508984/> | Out of Scope | 9 |
| 100 | 10 | Child maltreatment prevention: a systematic review of reviews | Mikton et al. 2009 | 19551253 | <https://pubmed.ncbi.nlm.nih.gov/19551253/> | Out of Scope | 10 |
| 101 | 11 | Decision aids for people facing health treatment or screening decisions | Stacey et al. 2014 | 24470076 | <https://pubmed.ncbi.nlm.nih.gov/24470076/> | Out of Scope | 11 |
| 102 | 12 | Effects of computerized clinical decision support systems on practitioner performance and patient outcomes: a systematic review | Garg et al. 2005 | 15755945 | <https://pubmed.ncbi.nlm.nih.gov/15755945/> | Out of Scope | 12 |
| 103 | 13 | Estimations of worldwide prevalence of chronic hepatitis B virus infection: a systematic review of data published between 1965 and 2013 | Schweitzer et al. 2015 | 26231459 | <https://pubmed.ncbi.nlm.nih.gov/26231459/> | Out of Scope | 13 |
| 104 | 14 | Gaps between knowing and doing: understanding and assessing the barriers to optimal health care | Cochrane et al. 2007 | 17576625 | <https://pubmed.ncbi.nlm.nih.gov/17576625/> | Out of Scope | 14 |

| 105 | 15 | Global, regional, and national incidence, prevalence, and years lived with disability for 310 diseases and injuries, 1990–2015: a systematic analysis for the Global Burden of Disease Study 2015 | Vos et al. 2016 | 27733282 | <https://pubmed.ncbi.nlm.nih.gov/27733282/> | Out of Scope | 15 |
| --- | --- | --- | --- | --- | --- | --- | --- |
| 106 | 16 | Hepatotoxicity of Nonsteroidal Anti-Inflammatory Drugs: A Systematic Review of Randomized Controlled Trials | Sriuttha et al. 2018 | 29568654 | <https://pubmed.ncbi.nlm.nih.gov/29568654/> | Out of Scope | 16 |
| 107 | 17 | How is radiating leg pain defined in randomized controlled trials of conservative treatments in primary care? A systematic review | Lin et al. 2013 | 23939653 | <https://pubmed.ncbi.nlm.nih.gov/23939653/> | Out of Scope | 17 |
| 108 | 18 | Integrating Bodies of Evidence: Existing Systematic Reviews and Primary Studies | Robinson et al. 2015 | 25834891 | <https://pubmed.ncbi.nlm.nih.gov/25834891/> | Out of Scope | 18 |
| 109 | 19 | Interventions to modify health care provider adherence to asthma guidelines: a systematic review | Okelo et al. 2013 | 23979092 | <https://pubmed.ncbi.nlm.nih.gov/23979092/> | Out of Scope | 19 |
| 110 | 20 | Partial updating of clinical practice guidelines often makes more sense than full updating: a systematic review on methods and the development of an updating procedure | Becker et al. 2013 | 24125894 | <https://pubmed.ncbi.nlm.nih.gov/24125894/> | Out of Scope | 20 |
| 111 | 21 | Percutaneous Endoscopic Debridement and Drainage for Spinal Infection: Systemic Review and Meta- Analysis | Mao et al. 2019 | 31337161 | <https://pubmed.ncbi.nlm.nih.gov/31337161/> | Out of Scope | 21 |
| 112 | 22 | Systematic review of knowledge translation strategies in the allied health professions | Scott et al. 2012 | 22831550 | <https://pubmed.ncbi.nlm.nih.gov/22831550/> | Out of Scope | 22 |
| 113 | 23 | The diagnostic accuracy of the Kemp's test: a systematic review | Stuber et al. 2014 | 25202153 | <https://pubmed.ncbi.nlm.nih.gov/25202153/> | Out of Scope | 23 |
| 114 | 24 | The effect of English-language restriction on systematic review-based meta-analyses: a systematic review of empirical studies | Morrison et al. 2012 | 22559755 | <https://pubmed.ncbi.nlm.nih.gov/22559755/> | Out of Scope | 24 |
| 115 | 25 | The epidemiology of chronic pain in children and adolescents revisited: a systematic review | King et al. 2011 | 22078064 | <https://pubmed.ncbi.nlm.nih.gov/22078064/> | Out of Scope | 25 |
| 116 | 26 | The inclusion of reports of randomised trials published in languages other than English in systematic reviews | Moher et al. 2003 | 14670218 | <https://pubmed.ncbi.nlm.nih.gov/14670218/> | Out of Scope | 26 |
| 117 | 27 | The prevalence of musculoskeletal symptoms in the construction industry: a systematic review and meta- analysis | Umer et al. 2017 | 29090335 | <https://pubmed.ncbi.nlm.nih.gov/29090335/> | Out of Scope | 27 |
| 118 | 28 | The prevalence of neck pain in the world population: a systematic critical review of the literature | Fejer et al. 2005 | 15999284 | <https://pubmed.ncbi.nlm.nih.gov/15999284/> | Out of Scope | 28 |
| 119 | 29 | Tools developed and disseminated by guideline producers to promote the uptake of their guidelines | Flodgren et al. 2016 | 27546228 | <https://pubmed.ncbi.nlm.nih.gov/27546228/> | Out of Scope | 29 |
| 120 | 30 | Years lived with disability (YLDs) for 1160 sequelae of 289 diseases and injuries 1990-2010: a systematic analysis for the Global Burden of Disease Study 2010 | Vos et al. 2012 | 23245607 | <https://pubmed.ncbi.nlm.nih.gov/23245607/> | Out of Scope | 30 |
| 121 | 31 | Clinical course of pain and disability following primary lumbar discectomy: systematic review and meta-analysis | Rushton et al. 2020 | 31916000 | <https://pubmed.ncbi.nlm.nih.gov/31916000/> | Not Treatment | 1 |
| 122 | 32 | Clinical diagnosis scale for pain lumbar of facet origin: systematic review of literature and pilot study | Vega et al. 2018 | 29910103 | <https://pubmed.ncbi.nlm.nih.gov/29910103/> | Not Treatment | 2 |
| 123 | 33 | The impact of low back-related leg pain on outcomes as compared with low back pain alone: a systematic review of the literature | Konstantinou et al. 2013 | 23328336 | <https://pubmed.ncbi.nlm.nih.gov/23328336/> | Not Treatment | 3 |
| 124 | 34 | What are patient beliefs and perceptions about exercise for nonspecific chronic low back pain? A systematic review of qualitative studies | Slade et al. 2014 | 24300225 | <https://pubmed.ncbi.nlm.nih.gov/24300225/> | Not Treatment | 4 |
| 125 | 35 | Value-based care in the management of spinal disorders: a systematic review of cost-utility analysis | Indrakanti et al. 2012 | 22042716 | <https://pubmed.ncbi.nlm.nih.gov/22042716/> | Not Treatment | 5 |
| 126 | 36 | Incidence and prognosis of mid-back pain in the general population: A systematic review | Johansson et al. 2016 | 27146481 | <https://pubmed.ncbi.nlm.nih.gov/27146481/> | Not Treatment | 6 |

| 127 | 37 | Systematic review of lumbar provocation discography in asymptomatic subjects with a meta-analysis of false-positive rates | Wolfer et al. 2008 | 18690280 | <https://pubmed.ncbi.nlm.nih.gov/18690280/> | Not Treatment | 7 |
| --- | --- | --- | --- | --- | --- | --- | --- |
| 128 | 38 | A Best-Evidence Systematic Appraisal of the Diagnostic Accuracy and Utility of Facet (Zygapophysial) Joint Injections in Chronic Spinal Pain | Boswell et al. 2015 | 26218947 | <https://pubmed.ncbi.nlm.nih.gov/26218947/> | Not Treatment | 8 |
| 129 | 39 | Most red flags for malignancy in low back pain guidelines lack empirical support: a systematic review | Verhagen et al. 2017 | 28708761 | <https://pubmed.ncbi.nlm.nih.gov/28708761/> | Not Treatment | 9 |
| 130 | 40 | Particulate and non-particulate steroids in spinal epidurals: a systematic review and meta-analysis | Feeley et al. 2016 | 26873103 | <https://pubmed.ncbi.nlm.nih.gov/26873103/> | Not Treatment | 10 |
| 131 | 41 | Red flags to screen for malignancy and fracture in patients with low back pain | Downie et al. 2014 | 25257627 | <https://pubmed.ncbi.nlm.nih.gov/25257627/> | Not Treatment | 11 |
| 132 | 42 | Systematic Review of the Efficacy of Particulate Versus Nonparticulate Corticosteroids in Epidural Injections | Mehta et al. 2016 | 27915069 | <https://pubmed.ncbi.nlm.nih.gov/27915069/> | Not Treatment | 12 |
| 133 | 43 | An Update of the Systematic Appraisal of the Accuracy and Utility of Discography in Chronic Spinal Pain | Manchikanti et al. 2018 | 29565943 | <https://pubmed.ncbi.nlm.nih.gov/29565943/> | Not Treatment | 13 |
| 134 | 44 | A systematic review of low back pain and sciatica patients' expectations and experiences of health care | Hopayian et al. 2014 | 24787355 | <https://pubmed.ncbi.nlm.nih.gov/24787355/> | Not Treatment | 14 |
| 135 | 45 | Acetaminophen for Chronic Pain: A Systematic Review on Efficacy | Ennis et al. 2015 | 26572078 | <https://pubmed.ncbi.nlm.nih.gov/26572078/> | Not Treatment | 15 |
| 136 | 46 | Acute low back pain: systematic review of its prognosis | Pengel et al. 2003 | 12907487 | <https://pubmed.ncbi.nlm.nih.gov/12907487/> | Not Treatment | 16 |
| 137 | 47 | Clinical classification in low back pain: best-evidence diagnostic rules based on systematic reviews | Petersen et al. 2017 | 28499364 | <https://pubmed.ncbi.nlm.nih.gov/28499364/> | Not Treatment | 17 |
| 138 | 48 | Imaging strategies for low-back pain: systematic review and meta-analysis | Chou et al. 2009 | 19200918 | <https://pubmed.ncbi.nlm.nih.gov/19200918/> | Not Treatment | 18 |
| 139 | 49 | Measurement Properties of the Central Sensitization Inventory: A Systematic Review | Scerbo et al. 2017 | 28851012 | <https://pubmed.ncbi.nlm.nih.gov/28851012/> | Not Treatment | 19 |
| 140 | 50 | Prevalence of Recurrent Herniation Following Percutaneous Endoscopic Lumbar Discectomy: A Meta-Analysis | Yin et al. 2018 | 30045591 | <https://pubmed.ncbi.nlm.nih.gov/30045591/> | Not Treatment | 20 |
| 141 | 51 | Systematic literature review of imaging features of spinal degeneration in asymptomatic populations | Brinjikji et al. 2014 | 25430861 | <https://pubmed.ncbi.nlm.nih.gov/25430861/> | Not Treatment | 21 |
| 142 | 52 | Systematic review of patient history and physical examination to diagnose chronic low back pain originating from the facet joints | Maas et al. 2016 | 27723170 | <https://pubmed.ncbi.nlm.nih.gov/27723170/> | Not Treatment | 22 |
| 143 | 53 | Systematic Review of Prognostic Factors for Return to Work in Workers with Sub Acute and Chronic Low Back Pain | Steenstra et al. 2017 | 27647141 | <https://pubmed.ncbi.nlm.nih.gov/27647141/> | Not Treatment | 23 |
| 144 | 54 | Systematic review of tests to identify the disc, SIJ or facet joint as the source of low back pain | Hancock et al. 2007 | 17566796 | <https://pubmed.ncbi.nlm.nih.gov/17566796/> | Not Treatment | 24 |
| 145 | 55 | A systematic review comparing the costs of chiropractic care to other interventions for spine pain in the United States | Dageais et al. 2015 | 26482271 | <https://pubmed.ncbi.nlm.nih.gov/26482271/> | Not Treatment | 25 |
| 146 | 56 | A systematic review of cost-effectiveness modeling of pharmaceutical therapies in neuropathic pain: variation in practice, key challenges, and recommendations for the future | Critchlow et al. 2016 | 27563752 | <https://pubmed.ncbi.nlm.nih.gov/27563752/> | Not Treatment | 26 |
| 147 | 57 | A Systematic Review of Interventions and Programs Targeting Appropriate Prescribing of Opioids | Moride et al. 2019 | 31151331 | <https://pubmed.ncbi.nlm.nih.gov/31151331/> | Not Treatment | 27 |
| 148 | 58 | A systematic review of low back pain cost of illness studies in the United States and internationally | Dagenais et al. 2007 | 18164449 | <https://pubmed.ncbi.nlm.nih.gov/18164449/> | Not Treatment | 28 |
| 149 | 59 | A systematic review of the global prevalence of low back pain | Hoy et al. 2012 | 22231424 | <https://pubmed.ncbi.nlm.nih.gov/22231424/> | Not Treatment | 29 |

| 150 | 60 | Accuracy of clinical neurological examination in diagnosing lumbo-sacral radiculopathy: a systematic literature review | Tawa et al. 2017 | 28231784 | <https://pubmed.ncbi.nlm.nih.gov/28231784/> | Not Treatment | 30 |
| --- | --- | --- | --- | --- | --- | --- | --- |
| 151 | 61 | Cost-effectiveness of conservative treatments for neck pain: a systematic review on economic evaluations | Driessen et al. 2012 | 22447407 | <https://pubmed.ncbi.nlm.nih.gov/22447407/> | Not Treatment | 31 |
| 152 | 62 | Cost-effectiveness of general practice care for low back pain: a systematic review | Lin et al. 2011 | 21203890 | <https://pubmed.ncbi.nlm.nih.gov/21203890/> | Not Treatment | 32 |
| 153 | 63 | Cost-Effectiveness of Non-Invasive and Non- Pharmacological Interventions for Low Back Pain: a Systematic Literature Review | Andronis et al. 2017 | 27550240 | <https://pubmed.ncbi.nlm.nih.gov/27550240/> | Not Treatment | 33 |
| 154 | 64 | Cost-utility analyses in spine care: a qualitative and systematic review | Nwachukwu et al. 2015 | 25341977 | <https://pubmed.ncbi.nlm.nih.gov/25341977/> | Not Treatment | 34 |
| 155 | 65 | Cost-utility analysis in spine care: a systematic review | Kepler et al. 2011 | 22784806 | <https://pubmed.ncbi.nlm.nih.gov/22784806/> | Not Treatment | 35 |
| 156 | 66 | Current Evidence for Diagnosis of Common Conditions Causing Low Back Pain: Systematic Review and Standardized Terminology | Vining et al. 2019 | 31870637 | <https://pubmed.ncbi.nlm.nih.gov/31870637/> | Not Treatment | 36 |
| 157 | 67 | Diagnostic accuracy of self-report and subjective history in the diagnosis of low back pain with non- specific lower extremity symptoms: A systematic review | Shultz et al. 2014 | 25231775 | <https://pubmed.ncbi.nlm.nih.gov/25231775/> | Not Treatment | 37 |
| 158 | 68 | Factors defining care-seeking in low back pain--a meta-analysis of population based surveys | Ferreira et al. 2009 | 20036168 | <https://pubmed.ncbi.nlm.nih.gov/20036168/> | Not Treatment | 38 |
| 159 | 69 | Incidence of Surgical Site Infection After Spine Surgery: A Systematic Review and Meta-analysis | Zhou et al. 2020 | 31464972 | <https://pubmed.ncbi.nlm.nih.gov/31464972/> | Not Treatment | 39 |
| 160 | 70 | The prevalence of low back pain in the elderly. A systematic review of the literature | Bressler et al. 1999 | 10488512 | <https://pubmed.ncbi.nlm.nih.gov/10488512/> | Not Treatment | 40 |
| 161 | 71 | The prevalence of low back pain: a systematic review of the literature from 1966 to 1998 | B F Walker. 2000 | 10872758 | <https://pubmed.ncbi.nlm.nih.gov/10872758/> | Not Treatment | 41 |
| 162 | 72 | Efficacy of autologous platelet-rich plasma use for orthopaedic indications: a meta-analysis | Sheth et al. 2012 | 22241606 | <https://pubmed.ncbi.nlm.nih.gov/22241606/> | Not Specific to Low Back Pain | 1 |
| 163 | 73 | Role of kinesiophobia on pain, disability and quality of life in people suffering from chronic musculoskeletal pain: a systematic review | Luque-Suarez et al. 2018 | 29666064 | <https://pubmed.ncbi.nlm.nih.gov/29666064/> | Not Specific to Low Back Pain | 2 |
| 164 | 74 | The efficacy of duloxetine, non-steroidal anti- inflammatory drugs, and opioids in osteoarthritis: a systematic literature review and meta-analysis | Myers et al, 2014 | 24618328 | <https://pubmed.ncbi.nlm.nih.gov/24618328/> | Not Specific to Low Back Pain | 3 |
| 165 | 75 | Clinical Diagnostic Tests versus Medial Branch Blocks for Adults with Persisting Cervical Zygapophyseal Joint Pain: A Systematic Review and Meta-Analysis | Usunier et al. 2018 | 29755174 | <https://pubmed.ncbi.nlm.nih.gov/29755174/> | Not Specific to Low Back Pain | 4 |
| 166 | 76 | Effectiveness of non-steroidal anti-inflammatory drugs for the treatment of pain in knee and hip osteoarthritis: a network meta-analysis | Costa et al. 2017 | 28699595 | <https://pubmed.ncbi.nlm.nih.gov/28699595/> | Not Specific to Low Back Pain | 5 |
| 167 | 77 | Relative benefit-risk comparing diclofenac to other traditional non-steroidal anti-inflammatory drugs and cyclooxygenase-2 inhibitors in patients with osteoarthritis or rheumatoid arthritis: a network meta- analysis | Walsem et al. 2015 | 25879879 | <https://pubmed.ncbi.nlm.nih.gov/25879879/> | Not Specific to Low Back Pain | 6 |
| 168 | 78 | The Effectiveness and Risks of Fluoroscopically- Guided Cervical Medial Branch Thermal Radiofrequency Neurotomy: A Systematic Review with Comprehensive Analysis of the Published Data | Engel et al. 2016 | 26359589 | <https://pubmed.ncbi.nlm.nih.gov/26359589/> | Not Specific to Low Back Pain | 7 |
| 169 | 79 | A Model-Based Meta-analysis to Compare Efficacy and Tolerability of Tramadol and Tapentadol for the Treatment of Chronic Non-Malignant Pain | Mercier et al. 2014 | 25135386 | <https://pubmed.ncbi.nlm.nih.gov/25135386/> | Not Specific to Low Back Pain | 8 |
| 170 | 80 | A Systematic Overview of Reviews for Complementary and Alternative Therapies in the Treatment of the Fibromyalgia Syndrome | Lauche et al. 2015 | 26246841 | <https://pubmed.ncbi.nlm.nih.gov/26246841/> | Not Specific to Low Back Pain | 9 |

| 171 | 81 | Acutherapy for Knee Osteoarthritis Relief in the Elderly: A Systematic Review and Meta-Analysis | Gong et al. 2019 | 30906410 | <https://pubmed.ncbi.nlm.nih.gov/30906410/> | Not Specific to Low Back Pain | 10 |
| --- | --- | --- | --- | --- | --- | --- | --- |
| 172 | 82 | Comparative effectiveness of pharmacologic interventions for knee osteoarthritis: a systematic review and network meta-analysis | Bannuru et al. 2015 | 25560713 | <https://pubmed.ncbi.nlm.nih.gov/25560713/> | Not Specific to Low Back Pain | 11 |
| 173 | 83 | Comparative pain reduction of oral non-steroidal anti- inflammatory drugs and opioids for knee osteoarthritis: systematic analytic review | Smith et al. 2016 | 26844640 | <https://pubmed.ncbi.nlm.nih.gov/26844640/> | Not Specific to Low Back Pain | 12 |
| 174 | 84 | Comparing Verum and Sham Acupuncture in Fibromyalgia Syndrome: A Systematic Review and Meta-Analysis | Kim et al. 2019 | 31534469 | <https://pubmed.ncbi.nlm.nih.gov/31534469/> | Not Specific to Low Back Pain | 13 |
| 175 | 85 | Correlation between acupuncture dose and effectiveness in the treatment of knee osteoarthritis: a systematic review | Sun et al. 2019 | 31271300 | <https://pubmed.ncbi.nlm.nih.gov/31271300/> | Not Specific to Low Back Pain | 14 |
| 176 | 86 | Effectiveness of cervical epidural injections in the management of chronic neck and upper extremity pain | Diwan et al. 2012 | 22828692 | <https://pubmed.ncbi.nlm.nih.gov/22828692/> | Not Specific to Low Back Pain | 15 |
| 177 | 87 | Effects of non-pharmacological conservative treatment on pain, range of motion and physical function in patients with mild to moderate hip osteoarthritis. A systematic review | Ceballos-Laita et al. 2018 | 30670244 | <https://pubmed.ncbi.nlm.nih.gov/30670244/> | Not Specific to Low Back Pain | 16 |
| 178 | 88 | Effects of orthopaedic manual therapy in knee osteoarthritis: a systematic review and meta-analysis | Anwer et al. 2018 | 30030035 | <https://pubmed.ncbi.nlm.nih.gov/30030035/> | Not Specific to Low Back Pain | 17 |
| 179 | 89 | Effects of Physical-Agent Pain Relief Modalities for Fibromyalgia Patients: A Systematic Review and Meta-Analysis of Randomized Controlled Trials | Honda et al. 2018 | 30402199 | <https://pubmed.ncbi.nlm.nih.gov/30402199/> | Not Specific to Low Back Pain | 18 |
| 180 | 90 | Efficacy and Safety of Duloxetine on Osteoarthritis Knee Pain: A Meta-Analysis of Randomized Controlled Trials | Wang et al. 2015 | 26176791 | <https://pubmed.ncbi.nlm.nih.gov/26176791/> | Not Specific to Low Back Pain | 19 |
| 181 | 91 | Efficacy and Safety of Surgical Interventions for Treating Multilevel Cervical Spondylotic Myelopathy via Anterior Approach: A Network Meta-Analysis | Li et al. 2019 | 31337165 | <https://pubmed.ncbi.nlm.nih.gov/31337165/> | Not Specific to Low Back Pain | 20 |
| 182 | 92 | Efficacy of High-Intensity Laser Therapy in Treating Knee Osteoarthritis: A First Systematic Review | Wyszynska et al. 2018 | 29688827 | <https://pubmed.ncbi.nlm.nih.gov/29688827/> | Not Specific to Low Back Pain | 21 |
| 183 | 93 | Efficacy of low-level laser therapy on pain and disability in knee osteoarthritis: systematic review and meta-analysis of randomised placebo-controlled trials | Stausholm et al. 2019 | 31662383 | <https://pubmed.ncbi.nlm.nih.gov/31662383/> | Not Specific to Low Back Pain | 22 |
| 184 | 94 | Local anesthetic injections with or without steroid for chronic non-cancer pain: a protocol for a systematic review and meta-analysis of randomized controlled trials | Shanthanna et al. 2016 | 26831725 | <https://pubmed.ncbi.nlm.nih.gov/26831725/> | Not Specific to Low Back Pain | 23 |
| 185 | 95 | Low-Level Laser Therapy for Fibromyalgia: A Systematic Review and Meta-Analysis | Yeh et al. 2019 | 31151332 | <https://pubmed.ncbi.nlm.nih.gov/31151332/> | Not Specific to Low Back Pain | 24 |
| 186 | 96 | Manipulation and Mobilization for Treating Chronic Nonspecific Neck Pain: A Systematic Review and Meta-Analysis for an Appropriateness Panel | Coulter et al. 2019 | 30921975 | <https://pubmed.ncbi.nlm.nih.gov/30921975/> | Not Specific to Low Back Pain | 25 |
| 187 | 97 | Mindfulness Meditation for Primary Headache Pain: A Meta-Analysis | Gu et al. 2018 | 29578127 | <https://pubmed.ncbi.nlm.nih.gov/29578127/> | Not Specific to Low Back Pain | 26 |
| 188 | 98 | NSAIDs vs acetaminophen in knee and hip osteoarthritis: a systematic review regarding heterogeneity influencing the outcomes | Verkleij et al. 2011 | 21619937 | <https://pubmed.ncbi.nlm.nih.gov/21619937/> | Not Specific to Low Back Pain | 27 |
| 189 | 99 | Oral or transdermal opioids for osteoarthritis of the knee or hip | Costa et al. 2014 | 25229835 | <https://pubmed.ncbi.nlm.nih.gov/25229835/> | Not Specific to Low Back Pain | 28 |
| 190 | 100 | Physical risk factors for developing non-specific neck pain in office workers: a systematic review and meta- analysis | Jun et al. 2017 | 28224291 | <https://pubmed.ncbi.nlm.nih.gov/28224291/> | Not Specific to Low Back Pain | 29 |
| 191 | 101 | Posterior Cervical Foraminotomy Via Full-Endoscopic Versus Microendoscopic Approach for Radiculopathy: A Systematic Review and Meta-analysis | Wu et al. 2019 | 30700067 | <https://pubmed.ncbi.nlm.nih.gov/30700067/> | Not Specific to Low Back Pain | 30 |

| 192 | 102 | Systematic review of efficacy and safety of buprenorphine versus fentanyl or morphine in patients with chronic moderate to severe pain | Wolff et al. 2012 | 22443154 | <https://pubmed.ncbi.nlm.nih.gov/22443154/> | Not Specific to Low Back Pain | 31 |
| --- | --- | --- | --- | --- | --- | --- | --- |
| 193 | 103 | Systematic Review of Nondrug, Nonsurgical Treatment of Shoulder Conditions | Hawk et al. 2017 | 28554433 | <https://pubmed.ncbi.nlm.nih.gov/28554433/> | Not Specific to Low Back Pain | 32 |
| 194 | 104 | Systematic review of the effectiveness of cervical epidurals in the management of chronic neck pain | Benyamin et al. 2009 | 19165300 | <https://pubmed.ncbi.nlm.nih.gov/19165300/> | Not Specific to Low Back Pain | 33 |
