# Supplementary material for: Reporting and methodological quality of systematic reviews underpinning clinical practice guidelines for low back pain: a meta-epidemiological study
Source: Front Pain Res (Lausanne). 2025 Dec 3;6:1704833. doi: 10.3389/fpain.2025.1704833 (PMC12708511; doi:10.3389/fpain.2025.1704833)
Supplement: Supplementary file 9 [file Table9.docx]

| **Supplementary Table 6. Murad and Wang Checklist** |  |  |  |
| --- | --- | --- | --- |
| **Checklist Item** | **Status** | **Manuscript Location** | **Changes made to text** |
| Identify study as meta-epidemiological in title/abstract | Partial – add wording | Title; Abstract (Design line) | Added to Title and Abstract: “… A Meta-epidemiological Study.” |
| Protocol existence & public access | Meets | Methods (first paragraph) | No change needed |
| Eligibility criteria (with rationale) | Meets | Methods → Identification of CPGs | No change needed |
| Information sources & search dates | Meets | Methods → Identification of CPGs | No change needed |
| Full search strategy reproducible | Meets | Supplementary File 1,  Supplementary Table 5 | No change needed |
| Study selection (process; duplicate; tool) | Meets | Methods → Identification of CPGs | No change needed |
| Data collection/extraction (piloted; duplicate; masking) | Meets | Methods → Data Extraction and Scoring | No change needed |
| Data items (variables) explicitly listed | Partial – add sentence | Methods → Data Extraction and Scoring | Added paragraph discussing data items and variables |
| Risk of bias in individual primary studies | Clarified – not applicable | Methods → Data Extraction and Scoring | Added "Because the unit of analysis was the SR, risk-of- bias within primary studies was not collected; instead, methodological quality of SRs was appraised with AMSTAR- 2." |
| Summary measures & synthesis methods | Meets | Methods → Statistics; Results | No change needed |
| Additional analyses (subgroup/regression) described | Meets | Methods → Secondary Analysis and Statistics | Added paragraph |
| Flow of records with counts (diagram) | Meets | Results; Figure 1 (and/or Supplement) | No change needed |

| Inter-reviewer agreement reported or justified | Partial – add justification | Methods → Data Extraction and Scoring; Discussion → Limitations | Added to Methods and Discussion: “We did not calculate a formal inter- reviewer agreement statistic; we conducted pilot calibration and reconciled all disagreements by consensus, which we acknowledge as a limitation.” |
| --- | --- | --- | --- |
| Study characteristics & per-study data available | Meets | Results; Supplementary Tables S2–S4 | No change needed |
| Discussion situates findings; certainty/robustness stated | Partial – add 1 line | Discussion | Added paragraph to discussion |
| Limitations (methodological) transparently stated | Meets | Discussion → Strengths and Limitations | No change needed |
| Conclusions (implications for research/practice) | Meets | Discussion → Implications | No change needed |
| Funding/role of funders; conflicts | Meets | Funding; Conflicts of Interest | No change needed |
| Data/materials availability (OSF) | Meets | Data Availability; Methods (first paragraph) | No change needed |
| Transparency: screened-record log provided | Meets | Methods; Supplementary Table 5 | No change needed |
